# Unveiling the mechanisms of neuropathic pain suppression: perineural resiniferatoxin targets Trpv1 and beyond

**DOI:** 10.3389/fnana.2023.1306180

**Published:** 2023-11-30

**Authors:** Safa Shehab, Hayate Javed, Aishwarya Mary Johnson, Saeed Tariq, Challagandla Anil Kumar, Bright Starling Emerald

**Affiliations:** Department of Anatomy, College of Medicine and Health Sciences, United Arab Emirates University, Al-Ain, United Arab Emirates

**Keywords:** Trpv1, resiniferatoxin, nerve injury, ion channels, neuropathic pain

## Abstract

Neuropathic pain arises from damage or disorders affecting the somatosensory system. In rats, L5 nerve injury induces thermal and mechanical hypersensitivity/hyperalgesia. Recently, we demonstrated that applying resiniferatoxin (RTX) directly on uninjured L3 and L4 nerves alleviated thermal and mechanical hypersensitivity resulting from L5 nerve injury. Herein, using immunohistochemistry, Western blot, and qRT-PCR techniques, we reveal that perineural application of RTX (0.002%) on the L4 nerve substantially downregulated the expression of its receptor (Trpv1) and three different voltage-gated ion channels (Nav1.9, Kv4.3, and Cav2.2). These channels are found primarily in small-sized neurons and show significant colocalization with Trpv1 in the dorsal root ganglion (DRG). However, RTX treatment did not affect the expression of Kv1.1, Piezo2 (found in large-sized neurons without colocalization with Trpv1), and Kir4.1 (localized in satellite cells) in the ipsilateral DRGs. Furthermore, RTX application on L3 and L4 nerves reduced the activation of c-fos in the spinal neurons induced by heat stimulation. Subsequently, we investigated whether applying RTX to the L3 and L4 nerves 3 weeks before the L5 nerve injury could prevent the onset of neuropathic pain. Both 0.002 and 0.004% concentrations of RTX produced significant analgesic effects, while complete prevention of thermal and mechanical hypersensitivity required a concentration of 0.008%. Importantly, this preventive effect on neuropathic manifestations was not associated with nerve degeneration, as microscopic examination revealed no morphological changes. Overall, this study underscores the mechanisms and the significance of perineural RTX treatment applied to adjacent uninjured nerves in entirely preventing nerve injury-induced neuropathic pain in humans and animals.

## 1 Introduction

Neuropathic pain is caused by a lesion or disease of the peripheral somatosensory nervous system. Despite the advances in treating neuropathic pain with several drugs, one of the important challenges is the lack of adequate efficacy of currently available pharmacotherapy (Finnerup, 2019; van Velzen et al., 2020). However, one of the promising recent approaches to treat neuropathic pain is the use of capsaicin (the ingredient of hot chilli peppers) (Fattori et al., 2016; Iadarola et al., 2021). Local application of capsaicin has been shown to cause a burning sensation of pain in human skin and mucosa by activating its receptor, transient receptor potential vanilloid 1 (Trpv1) ion channel, while later the threshold of noxious stimuli will be increased, leading to desensitization (Jancśo et al., 1980; Szolcsányi, 2014). Consequently, capsaicin and its ultrapotent analog resiniferatoxin (RTX) have been widely used to treat pain in both clinical and preclinical studies (Brown, 2016; Fattori et al., 2016; Moran and Szallasi, 2018; Sapio et al., 2018; Iadarola et al., 2021).

In addition to the Trpv1, voltage-gated ion channels could be involved in nociception and neuropathic pain. Voltage-gated sodium channels (Nav) such as Nav1.1, Nav1.6, Nav1.7, Nav1.8, and Nav1.9 are all expressed by adult dorsal root ganglion (DRG) neurons, which might contribute to the sensitization of sensory neurons in chronic pain (Stevens and Stephens, 2018; Bennett et al., 2019; McDermott et al., 2019). Given the important role of voltage-gated potassium (Kv) channels in limiting neuronal excitability, the number of subclasses (Kv1.1, 3.3, 3.4, 4.1, 4.2, 4.3, and 9.1) have been identified in the sensory neurons with evidence that their dysfunction may play a role in the onset and maintenance of neuropathic pain (Rasband et al., 2001; Chien et al., 2007; Tsantoulas et al., 2012; Tsantoulas and McMahon, 2014; Smith, 2020; Kanda et al., 2021). Furthermore, voltage-gated calcium channels (Cavs) have also been shown to play an important role in neuropathic pain (Luo et al., 2001; Feng et al., 2019). Low-voltage activated Cavs are of three subtypes based on the pore-forming α1 subunit: Cav3.1, Cav3.2, and Cav3.3 (Catterall et al., 2005; Iftinca, 2011).

Neuropathic pain represents a pathological response of injury to the somatosensory system and, consequently most animal models involve experimental injury to peripheral nerves (Ossipov and Porreca, 2013). Spinal nerve ligation (SNL) is the most commonly used model of neuropathic pain in rodents in which the fifth lumbar (L5) or L5 and L6 spinal nerves are ligated and cut distally (Kim and Chung, 1992). The animals in this model would show hyperalgesia and allodynia as seen in humans (Kim and Chung, 1992). To further understand the mechanisms of neuropathic pain in this model, our anatomical findings showed the spinal terminations of unmyelinated primary afferents of L4 and L5 spinal nerves overlap in the dorsal horn at the L3-L5 spinal levels (Shehab et al., 2015). Based on the fact that hyperalgesia and allodynia require an intact nerve to conduct the noxious and tactile information from the hind paw skin to the spinal cord, we provided anatomical evidence that in cases where the L5 nerve has been injured in rats, the adjacent uninjured L4 nerve would be the most likely candidate to mediate the resulting neuropathic pain (Shehab et al., 2015). Furthermore, we have shown earlier that chemical inactivation of intact L3 and L4 nerves with RTX (0.002%) suppressed the neuropathic pain induced by adjacent L5 nerve injury (Javed et al., 2020). The decrease in TRPV1 expression following perineural application of RTX may elucidate the reduction and elimination of thermal hypersensitivity/hyperalgesia (Javed et al., 2020). However, it is worth considering that this RTX treatment in reducing the mechanical hyperalgesia may be linked to the downregulation of other nociceptive molecules that coexist with TRPV1 neurons in the DRG.

The objectives of this study were to (1) show evidence that in addition to its effects on Trpv1, the perineural application of RTX would also cause down-regulation of the expression of Nav1.9, Kv4.3, and Cav2.2, which are found in small sensory and nociceptive neurons in the DRG. This, in turn, would decrease the excitability of DRG neurons, reducing both thermal and mechanical hyperalgesia. In this regard, we first determined the localization and colocalization of TRPV1 with Nav1.9, Kv4.3 and Cav2.2 in small sensory neurons of the DRG. As a control experiment, Kv1.1 and Piezo2 were used as markers of large proprioceptive/mechanoreceptive neurons and Kir4.1 as a marker for satellite cells in the DRG. We hypothesized that RTX treatment would cause downregulation of its receptor (Trpv1) and those ion channels that colocalised without any significant effects on Kv1.1, Piezo2 and Kir4.1. (2) find out whether perineural application of the appropriate dose of RTX on uninjured L3 and L4 nerves would not only produce significant reduction but completely prevent L5 nerve-injury induced thermal and mechanical hypersensitivity. To achieve that, we applied RTX on L3 and L4 nerves in rats, and after three weeks, the L5 nerve was injured to induce neuropathic pain. The responses to the thermal and mechanical stimuli were tested three weeks before and 3–28 days after the L5 nerve injury. (3) determine whether the appropriate dose of RTX, which could completely prevent the development of neuropathic pain, would be due to peripheral nerve degeneration.

## 2 Materials and methods

### 2.1 Animals

Male Wistar rats were bred and maintained at the animal research facility of the College of Medicine and Health Sciences (CMHS) at the United Arab Emirates (UAE) University. Animals were maintained on a 12-h dark/light cycle with food and water *ad libitum*. All experimental procedures were approved by the Animal Ethics Committee of the CMHS, UAE University (ERA-2020-7222) and were performed in accordance with the guidelines of the European Communities Council Directive of November 24, 1986 (86/609/EEC).

### 2.2 Resiniferatoxin (RTX) administration, surgery, and behavioral testing

Rats (weighing 240–255 g) were acclimatized in the behavioral testing chambers located on a glass platform or metal mesh for thermal or mechanical hypersensitivity, respectively, for at least 2 h every day for 3 days and then measured for baseline values. Subsequently, rats were anesthetized with 2–2.5% isoflurane in an air mixture administered through a nose cone. The skin of the back was incised longitudinally, the transverse processes of the fifth lumbar vertebrae were excised, and the L4 nerve was exposed. The L3 nerve was also exposed by teasing a small portion of muscle located lateral to the L4 nerve. Subsequently, RTX (Sigma, St. Louis, MO, USA) either 2 μg (0.002%) or 4 μg (0.004%) or 8 μg (0.008%) or vehicle (10% Tween 80, 10% EtOH, 80% normal saline) in a volume of 75 μL was applied perineurally on the L4 and L3 nerves for 30 min under anaesthesia. Then, the muscles and the skin were sutured in layers, and rats were given appropriate postoperative care. After 3, 7, 14 and 21 days of RTX/vehicle application, rats were tested in response to thermal and mechanical stimuli. Subsequently, rats underwent a second surgery for L5 spinal nerve ligation, as reported previously (Kim and Chung, 1992; Shehab et al., 2014; Javed et al., 2020). The left L5 nerve was ligated with a 6/0 silk suture and sectioned distally. The muscles and skin of the back were sutured in layers, and rats received sufficient postoperative care. To observe the analgesic effects of different doses of perineurally applied RTX, rats were again tested for thermal and mechanical hyperalgesia at different time points 3,7,14,21 and 28 days after L5 nerve injury.

The behavioral tests were performed between 8:00 and 16:00 h. The thermal and mechanical thresholds of paw withdrawal were assessed using a plantar and dynamic plantar aesthesiometer, respectively (Ugo Basile, Italy). The withdrawal latency for thermal hypersensitivity was recorded by a digital timer connected to a mobile radiant heat source located underneath the glass that was focused on one hind paw. The infrared stimulus was set at 70 a.u. with a cut-off latency of 20 s to prevent tissue damage. Mechanical hypersensitivity was measured with a movable force actuator positioned below the plantar surface of the animal, and the desired force and force speed were applied at 2.5 g/s. A Von Frey-type 0.5-mm filament exerted force incrementally until the animal briskly withdrew the hind paw. At each paw withdrawal, a digital recorder connected to the movable force actuator recorded the latency time and the actual force applied during the paw withdrawal reflex. The investigator who performed the behavioral tests was blinded to the types of treatment and groups.

### 2.3 Immunohistochemistry

RTX (0.002%) or vehicle 75 μL was applied perineurally on the L4 nerve, and after 2 weeks, the animals were perfused. Briefly, the animals were anesthetized with sevoflurane and perfused through the ascending aorta with 4% paraformaldehyde in 0.1 M phosphate buffer (pH 7.4). The fourth ipsilateral and contralateral DRGs and nerves were dissected and post-fixed for 1 h. Then, all DRGs were stored in 30% sucrose in phosphate buffer overnight at 4 ° C.

#### 2.3.1 DRGs staining

Serial cryostat sections of L4 DRG (15 μm thick) were collected from the ipsilateral and contralateral sides on gelatin-coated slides. The sections were then incubated overnight in a set of double immunofluorescent staining with a mixture of primary antibodies, including guinea pig anti-Trpv1, rabbit anti-Nav1.9, rabbit anti-Kv4.3, rabbit anti-Cav2.2, rabbit anti-Kv1.1, rabbit anti-Piezo2, and rabbit anti-Kir4.1. After washing, sections were incubated for 1 h in appropriate species-specific secondary antibodies (Donkey anti-guinea pig conjugated to Alexa 488 and anti-rabbit conjugated to Rhodamine Red). The sections were then counterstained with 4′,6-diamidino-2-phenylindole, di-hydrochloride (DAPI, Molecular Probes, Life Technologies, CA) for nuclear staining.

#### 2.3.2 Nerve staining

In addition, the ipsilateral and contralateral L4 nerves were sectioned (15 μm thick) using a cryostat and collected on gelatin-coated slides. Sections were triple labeled in two different sets. In the first set, the sections were incubated with a mixture of primary antibodies, including guinea pig anti-Trpv1, rabbit anti-Kv1.1, and mouse anti-NF200. In the second set, sections were incubated with guinea pig anti-Trpv1, goat anti-IB4 and mouse anti-NF200. For IB4 staining, sections were preincubated with IB4 antigen for 1 h. After washing, the sections were incubated for 1 h in appropriate species-specific secondary antibodies (Donkey anti-guinea pig conjugated to Alexa 488, anti-rabbit conjugated to Rhodamine Red or anti-goat conjugated to Rhodamine Red, and anti-mouse conjugated to Cy5).

### 2.4 Imaging and data analysis

Sections were examined with a Nikon fluorescent microscope (Nikon, Tokyo, Japan) equipped with appropriate filters to reveal Alexa 488 (green fluorescent) and Rhodamine Red (red fluorescent) or a Nikon C1 laser scanning confocal microscope to reveal Alexa 488, Rhodamine Red, and Cy5 (blue fluorescent) labeling. Representative digital images were captured using either a Nikon DS-Ri2 camera or a Nikon C1 confocal microscope. The resulting files were used to generate figures in Adobe Photoshop CS6 (San Jose, CA), where photomicrographs were adjusted for contrast and brightness.

### 2.5 Dorsal root ganglion

The quantification of ion channels labeled cells and its colocalization with Trpv1 was carried out in the ipsilateral and contralateral DRGs from animals treated with 0.002% of RTX or vehicle on the L4 nerves. From the serially sectioned DRGs, counting was performed in every ninth serial section (4–5 sections per DRG). All DRG sections were counterstained with DAPI to determine the percentage of neurons labeled with Trpv1, Kv1.1, Kv4.3, Nav1.9, Cav2.2, and Piezo2 in relation to the total number of neurons in each DRG. The colocalization of these markers in the individual cells was confirmed by the Neurolucida software (MBF Biosciences, Williston, VT). The percentage of individual markers was calculated by dividing the total number of DAPI-positive (DAPI+) neurons that contain respective markers by the total number of DAPI+ neurons and multiplying the result by 100. The percentage of double-labeled neurons was also calculated. In addition, Neurolucida software was used to measure the perimeter of neurons, which were labeled with Trpv1, Nav1.9, Kv4.3, Cav2.2, Kv1.1, and Piezo2 from 4–5 sections/per L4 DRG (n = 3). The perimeter of only labeled neurons with clear DAPI+ nuclei was measured.

### 2.6 c-fos staining in the spinal cord (ABC method)

To assess the effects of the perineural application of RTX on the pain marker c-fos, RTX (0.002%) or vehicle applied on the L4 and L3 nerves and L5 nerve injury was induced. After 3 weeks of RTX treatment and L5 injury, both hind paws of rats were immersed in a hot water bath at 50°C temperature for 2 min continuously under isoflurane anesthesia. The animal’s hind paws were carefully observed and showed no sign of burn injury. After 1 h 30 min of heat treatment, the rats were perfused as described above. L3 and L4 spinal segments were dissected out and post-fixed for 3–4 h and subsequently transferred to 30% sucrose overnight at 4 ° C. Transverse cryostat sections (50 μm) of L3 and L4 spinal segments were prepared and processed for immunohistochemistry. The sections were incubated overnight with rabbit anti-c-fos (1:5000) at room temperature. After rinsing with PBS, the sections were incubated with biotinylated anti-rabbit secondary antibody (1:500; Jackson Immuno Research, West Grove, PA) for an hour, followed by extravidin peroxidase conjugate for 1 h (1,1,000, Sigma-Aldrich). Finally, the sections were incubated for 4–5 min in a solution of 3,3′-diaminobenzidine (DAB) solution (25 mg / 50 mL of phosphate buffer, pH 7.4 with 7.5 μL hydrogen peroxide [30%] and 1 mL of nickel chloride [3%] added to it). All sections were mounted on gelatin-coated slides and allowed to air-dry overnight. They were then washed, dehydrated in graded alcohol, cleared in xylene, cover slipped, and examined under light microscope. The quantification of c-fos labeled nuclei was carried out in the medial half area of the dorsal horn of 8 random sections of the L3 and L4 spinal segments from each animal. Images were captured at 10x objective, and the number of nuclei was manually counted by Image J software.

Details of the primary antibodies used, their source, and dilutions are given in Table 1.

**Table 1:** Primary antibodies used, source and dilution.

Antibody	Immunogen	Host	Catalogue/source	RRIDs	Dilution IHC/WB
Trpv1	YTGSLKPEDAEVFKDSMVPGEK	Guinea Pig	GP14100/ Neuromics, MN, United States	AB_1624142	1:2000/−
Trpv1	Synthetic peptide corresponding to aa 824–838 (C-terminus) of rat Trpv1	Rabbit	BML SA-564/ ENZO Life Sciences, Postfach CH-4415 Lausen Switzerland		−/1:500
IB4	Purified *Griffonia simplicifolia lectin I* whole molecule	Goat	AS2104/Vector Laboratories, Peterborough, United Kingdom	AB_2314660	1:1000/−
Kv1.1	GST fusion protein with the sequence HRETEGEEQAQLLHV SSPNLASDSDLSRRSSSTISKSEYMEIEEDMNNSIAHYRQANIRTGNCTTADQNCVNKSKLLTDV, corresponding to amino acid residues 416–495 of mouse K_V_1.1	Rabbit	APC-009/Alomone Labs, Jerusalem BioPark (JBP) Jerusalem 9,104,201 Israel	AB_2040144	1:500/ 1:500
Kir4.1	Peptide (C)KLEE SLREQ AEKEG SALSV R, corresponding to amino acid residues 356–375 of rat K_ir_4.1	Rabbit	APC-035/Alomone Labs, Jerusalem BioPark (JBP) Jerusalem 9,104,201 Israel	AB_2040120	1:1000/ 1:500
Kv4.3	Peptide(C)NEALELTGTPEEEHMGK, corresponding to amino acid residues 451–468 of human K_V_4.3	Rabbit	APC-017/Alomone Labs, Jerusalem BioPark (JBP) Jerusalem 9,104,201 Israel	AB_2040178	1:1000/ 1:500
Nav1.9	Peptide CNGDLSSLDVAKVKVHND, corresponding to amino acid residues 1748–1765 of rat Na_V_1.9	Rabbit	ASC-017/Alomone Labs, Jerusalem BioPark (JBP) Jerusalem 9,104,201 Israel	AB_2040200	1:2000/ 1:500
Cav2.2	Peptide(C)RHHRHRDRDKTSASTPA, corresponding to amino acid residues 851–867 of rat CACNA1B	Rabbit	ACC-002/Alomone Labs, Jerusalem BioPark (JBP) Jerusalem 9,104,201 Israel	AB_2039766	1:1000/ 1:500
Piezo2	Peptide corresponding to 19 amino acids near the amino terminus of human PIEZO2	Rabbit	ProSci, 12,170 Flint Place Poway, CA 92064, United States		1:1000/ 1:500
c-fos	KLH-conjugated linear peptide corresponding to 14 amino acids from the *N*-terminal region of human c-fos	Rabbit	ABE457/Merck Millipore, MA, United States		1:5000/−

### 2.7 Quantitative real-time polymerase chain reaction

The mRNA expression levels of Trpv1, Nav1.9, Kv4.3, Cav2.2, Kv1.1, Piezo2 and Kir4.1 were quantified after 2 weeks of perineural application of RTX (0.002%) or vehicle on L4 nerve in rats. Total RNA was extracted from left L4 DRGs from RTX and vehicle-treated rats as well as right control L4 DRGs. The qRT-PCR analysis was performed as we published previously (Emerald et al., 2011; Javed et al., 2020, 2022). Briefly, total RNA was extracted using TRIzol™ Reagent (Thermo Fisher Scientific) according to the manufacturer’s instructions. Total RNA (1 μg) was converted to cDNA using the Applied Biosystems high-capacity cDNA reverse transcription kit (Applied Biosystems). PCR reactions were carried out in a volume of 20 μL of Luna® Universal qPCR Master Mix (New England BioLabs) with 200 ng of cDNA and 0.25 μM of each primer, using QuantStudio™ 3 real-time PCR System (Applied Biosystems). Relative gene expression was calculated using the comparative Ct method (Schmittgen and Livak, 2008). 18S RNA was used as an internal control.

The primers used were as follows:

**Table:** 

Gene	Forward	Reverse
Trpv1	CATGGGTG AGACCGTCAACA	AGGCCTTCCTCATGCACTTC
Kv1.1	GCCGCAGCTCCTCTACTATC	TTTGATTGCTTGCCTGGTGC
Kir4.1	CCACCTCTGTGCCAAGATGAC	AGGACCCTCCTCCGACGTAT
Kv4.3	CTCCAATGCCTACCTGCACA	GTTCGTACAGACAACAGGGGA
Nav1.9	CATACGGTGCCCTGATCCTC	CAGCCAGAGAGTCGGAAGTG
Cav2.2	AGTCCCCTTTGGATGCAGTG	CCTCCGGAAGTACGATGAGC
Piezo2	GGATGAAGAACCACCACGGA	GACGAACTTTGCAGCTCTGA
18S	AGTCCCTGCCCTTTGTACACA	GATCCGAGGG CCTCACTAAAC

### 2.8 Western blot

The protein expression level of Trpv1, Nav1.9, Kv4.3, Cav2.2, Kv1.1, Piezo2, and Kir4.1 was measured by western blotting after 2 weeks of perineural application of RTX 0.002% or vehicle on L4 nerve. Left L4 DRGs from RTX, and vehicle-treated rats as well as right control L4 DRGs, were homogenized in RIPA buffer with protease and phosphatase inhibitors as described previously (Emerald et al., 2011; Javed et al., 2020). The tissue lysates were then centrifuged at 15,000 rpm for 20 min. The supernatant was isolated, and protein concentration was quantified using the Pierce BCA protein assay kit (Thermo Fisher Scientific, Rockford, IL) following the manufacturer’s instructions. Subsequently, equal amounts of protein (30 μg) were loaded and separated using 4–12% SDS–polyacrylamide gel electrophoresis. The proteins were then transferred to a PVDF membrane and incubated overnight at 4 ° C with specific primary antibodies raised in rabbits against Trpv1, Nav1.9, Kv4.3, Cav2.2, Kv1.1, Piezo2 and Kir4.1 (1:500). The membrane was washed and then incubated with horseradish peroxidase-conjugated secondary anti-rabbit antibody. The protein recognized by the antibody was visualized using an enhanced chemiluminescence Pico kit (Thermo Fisher Scientific, Rockford, IL). As a loading control, the blots were stripped and re-probed for *β*-actin (1:5,000; monoclonal mouse, Millipore, MA). The intensity of the bands was measured using densitometry and quantified using Image J software (NIH, Bethesda, MD).

### 2.9 Transmission Electron microscopy (TEM) examination

TEM was used to examine the ultrastructural changes in DRG and unmyelinated axons following the perineural application of 0.008% RTX on the L4 nerve. After 14 days of RTX application, rats were anesthetized with sevoflurane and perfused with Karnovsky’s fixative (2% paraformaldehyde and 2.5% glutaraldehyde in phosphate buffer at 7.3 pH) through the ascending aorta. Thereafter, ipsilateral and contralateral L4 nerve and L4 DRG were dissected and post-fixed in the same fixative overnight at 4°C. Tissues were washed thrice with phosphate buffer (0.1 M, pH 7.4), each lasting 30 min. The tissues were then post-fixed in 0.5% osmium tetraoxide for 2 h, dehydrated in a series of graded ethanol, and finally with propylene oxide. The tissues were dipped in a mixture of Epoxy resin and propylene oxide in varying proportions and then infiltrated with 100% resin. Finally, the tissues were embedded in 100% freshly prepared resin and incubated at 65°C in an agar oven for 24 h. Semi-thin sections (1.5 μm) were acquired with a glass knife, and ultra-thin (95 nm) sections were acquired with a diamond knife using an ultramicrotome (Leica, EM UC 7, Vienna, Austria). Semi-thin sections were collected on glass slides and stained with 1% toluidine blue to reveal the overall structural morphology of DRG neurons and unmyelinated axons under a Nikon Eclipse Ci. Light microscope and images were acquired using a digital camera. Ultra-thin sections were placed on 100 Mesh Cu grids, and they were contrasted with 12% aqueous Uranyl acetate for 1 h and 4% Lead citrate as a double stain for 30 min. Ultra-thin sections were studied under a TEM (FEI, Tecnai G2 Spirit BioTwin TEM, Netherlands), and images were acquired for analysis.

#### 2.9.1 Ultrastructural observation and measurement of the perimeter (size) of unmyelinated axons following perineural application of RTX (0.008%) on the L4 nerve

To observe the ultrastructural changes in the unmyelinated axons of the L4 nerves and DRG neurons following perineural application of 0.008% RTX on the L4 nerve, a total of 20 micrographs per cross-sectional area (2 cross-sections) were prepared at magnifications up to 16,500×. Images of the L4 nerve (from both the ipsilateral and contralateral sides) were used to examine the unmyelinated axons and DRG neurons to confirm the injury, including swelling, disintegration, and dystrophy, with dark staining axoplasm, accumulation of abnormal membranous deposits, and destruction or degeneration of cellular organelles (Rambourg et al., 1983; Kissin et al., 2007; Javed et al., 2020). To examine the effect of RTX application on nerve fibers, the sizes of the unmyelinated axons of the L4 nerve in 20 micrographs per cross-section were acquired (randomly selected two sections/animal) at a magnification of 16,500× and were measured using Axio vision software (version 4.8; Carl Zeiss Microscopy, Jena, Germany). A total number of 1889, 1,550, and 1,568 axons were analyzed for the ipsilateral L4 nerve treated with RTX, the contralateral control L4 nerve and the L4 nerve treated with vehicle, respectively. The size measurement was performed by an investigator who was blind to the experimental groups.

### 2.10 Antibody characterization

According to the manufacturer’s technical information, the Trpv1 antibody selectively and specifically recognises Trpv1 of rat and mouse origin. We have previously characterized this antibody (Javed et al., 2020), and the pre-treatment of guinea pig anti-Trpv1 antibody with Trpv1 peptide (Neuromics, MN) at a concentration of 10^−5^ M completely abolished the positive staining compared with sections incubated with only Trpv1 antibody. In addition, this Trpv1 antibody showed negative labeling in the DRG of Trpv1−/− mice (Baiou et al., 2007). Similarly, incubation of rabbit anti-Trpv1 antibody pre-absorbed with Trpv1 control peptide (Cat No. BML-KI359, ENZO life Sciences) at a concentration of 10^−5^ M abolished the positive immunoreactivity revealed by incubation with only anti-Trpv1 antibody. The goat anti-IB4 antibody was produced against Griffonia simplicifolia lectin I as an immunogen. This antibody was purified using affinity chromatography on lectin-specific columns and yields identical recognition when tested by immunodiffusion (manufacturer’s technical information). Spinal cord sections of control rats, incubated with IB4 antibody, showed no immunoreactivity. Furthermore, the specificity of the IB4 antibody was shown by immunoreactivity in a specific area of the spinal cord (lamina II), while other areas did not show positive labeling (Shehab et al., 2014). In addition, sections that were not incubated with IB4 revealed no positive labeling. Pre-treatment of rabbit anti-Kv1.1, rabbit anti-Kir4.1, rabbit anti-Kv4.3, rabbit anti-Nav1.9 and rabbit anti-Cav2.2 with their respective immunogen (peptide) Kv1.1 (APC-161), Kir4.1 (APC-165), Kv4.3 (BLP-PC017), Nav1.9 (BLP-SC017) and Cav2.2 (BLP-CC002), respectively, at the molar concentration of 10^−5^ completely abolished positive labeling in the rat DRG sections. Furthermore, pre-treatment of rabbit anti-Piezo2 with the Pieoz2 peptide (8613P, ProSci) completely quenched the positive labeling of Piezo2 in DRG sections. The rabbit anti-c-fos antibody recognizes a band at 56 kDa. This antibody specifically reacts with human and rat tissues and is predicted to react with other species based on 100% sequence homology (manufacturer’s technical information). Furthermore, no immunoreactivity was detected in sections incubated with non-immune rabbit or goat serum.

### 2.11 Statistical analysis

The behavioral data are expressed as mean ± standard error of the mean (SEM) and were analyzed with Graph Pad (InStat software, La Jolla, CA) using a two-way analysis of variance (ANOVA) followed by Bonferroni *post hoc* test to determine the statistical significance between the means of various groups. Neuronal count data for Trpv1, Nav1.9, Kv4.3, Cav2.2, Kv1.1, and Piezo2 are expressed as percent mean ± SEM and analyzed using one-way ANOVA followed by the Bonferroni *post hoc* test. Furthermore, the qRT-PCR and Western blot data are statistically analyzed by one-way ANOVA followed by Tukey’s *post hoc* test. Student’s *t*-test was used to determine the significant differences in the sizes of the unmyelinated axons of the ipsilateral and contralateral L4 nerve result was considered statistically significant at *p* < 0.05.

## 3 Results

### 3.1 Localization and colocalization of Trpv1, Nav1.9, Kv4.3, Cav2.2, Kv1.1, Piezo2, and Kir4.1 in the DRG

**Figure 1:**
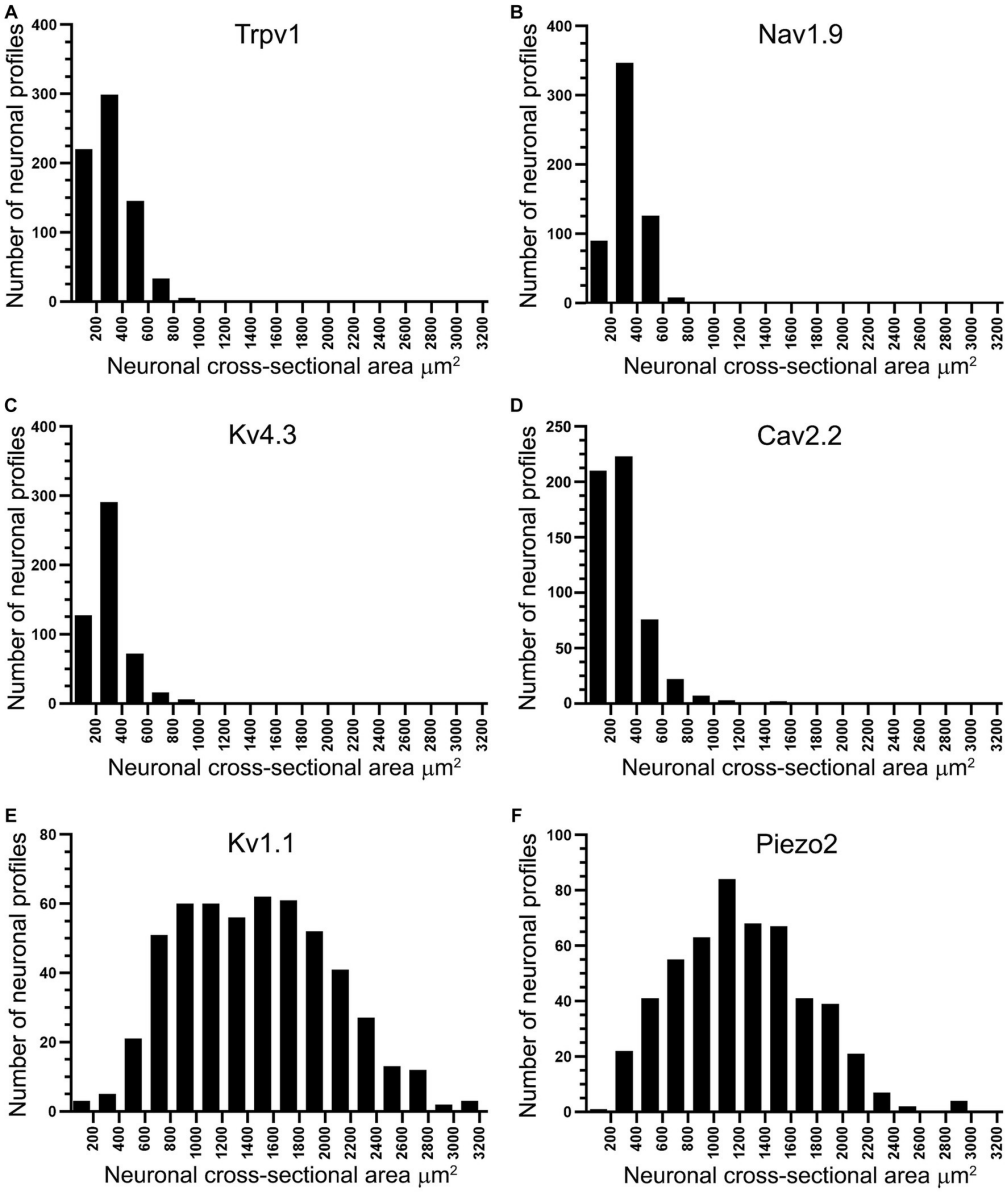
Histograms showing the cross-sectional area of neuron profiles in rat L4 DRG labeled with antibodies raised against Trpv1, Nav1.9, Kv4.3, Cav2.2, Kv1.1 and Piezo2. Note that Trpv1 **(A)**, Nav1.9 **(B)**, Kv4.3 **(C)**, and Cav2.2 **(D)** immunoreactive profiles were found in small-sized neurons, the majority of them smaller than 800 μm^2^. In contrast, Kv1.1 **(E)** and Piezo2 **(F)** immunoreactivities were found in larger neuronal profiles, the majority of them larger than 800 μm^2^.

**Figure 2:**
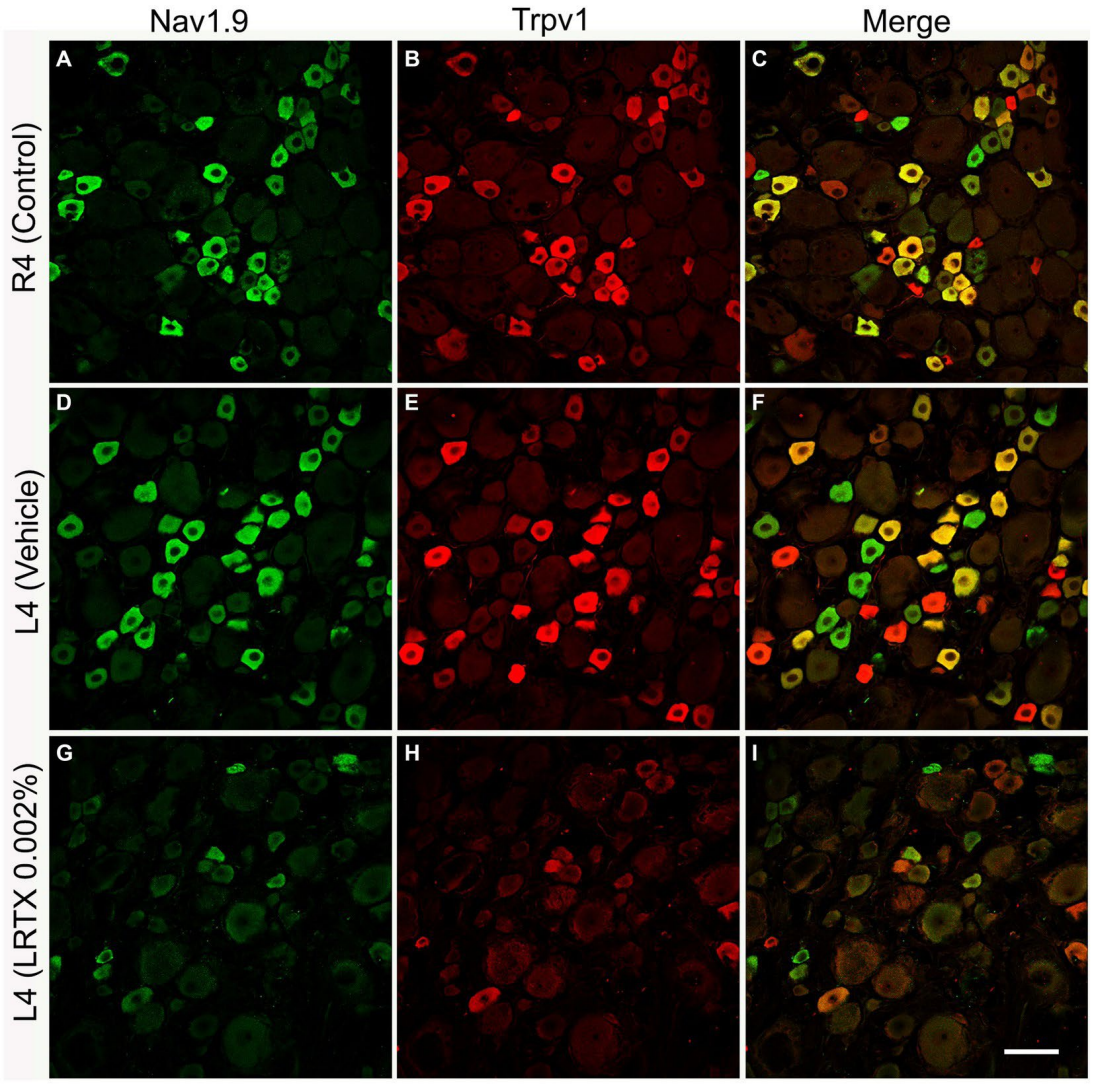
Illustrative images of double immunofluorescent labeling of Nav1.9 and Trpv1 in the control right L4 DRG (R4, **A–C**) and the left L4 DRG following the perineural application of either vehicle **(D–F)** or RTX (0.002%, G-I) on left L4 nerves. Both Nav1.9- **(A,D)** and Trpv1- **(B,E)** immunoreactivities were mainly found in small-sized neurons with significant colocalization **(C,F)**. RTX application on the L4 nerve caused a significant reduction in the number of Nav1.9- (**G**, *p < 0.05) and Trpv1- (**H**, ***p < 0.001) labeled neurons in the corresponding L4 DRG compared to the right control **(A,B)** and vehicle-treated L4 DRGs **(D,E)**. *n* = 4, scale bar = 50 μm.

**Figure 3:**
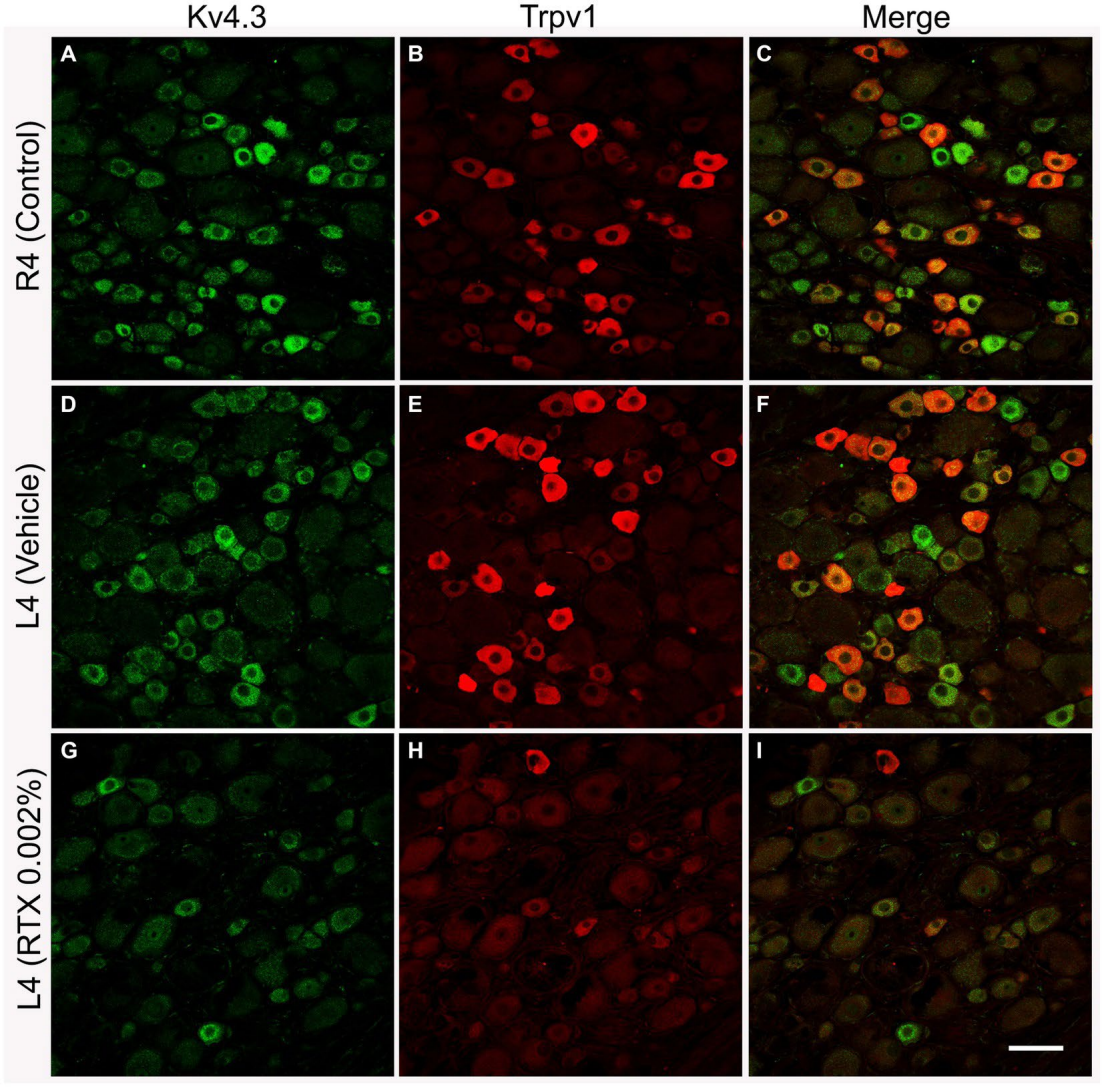
Representative images of double immunofluorescent labeling of Kv4.3 and Trpv1 in control right L4 DRG (R4, **A–C**) and the left L4 DRG following the perineural application of either vehicle **(D–F)** or RTX (0.002%, **G–I**) on L4 nerves. Both Kv4.3- **(A,D)** and Trpv1- **(B,E)** immunoreactivities were mainly found in small-sized neurons with a significant colocalization **(C,F)**. RTX application on the L4 nerve caused a significant reduction in the number of Kv4.3- (**G**, *p < 0.05) and Trpv1- (**H**, ***p < 0.001) labeled neurons in the corresponding L4 DRG compared to the right control **(A,B)** and vehicle-treated L4 DRGs **(D,E)**. *n* = 3–4, scale bar = 50 μm.

**Figure 4:**
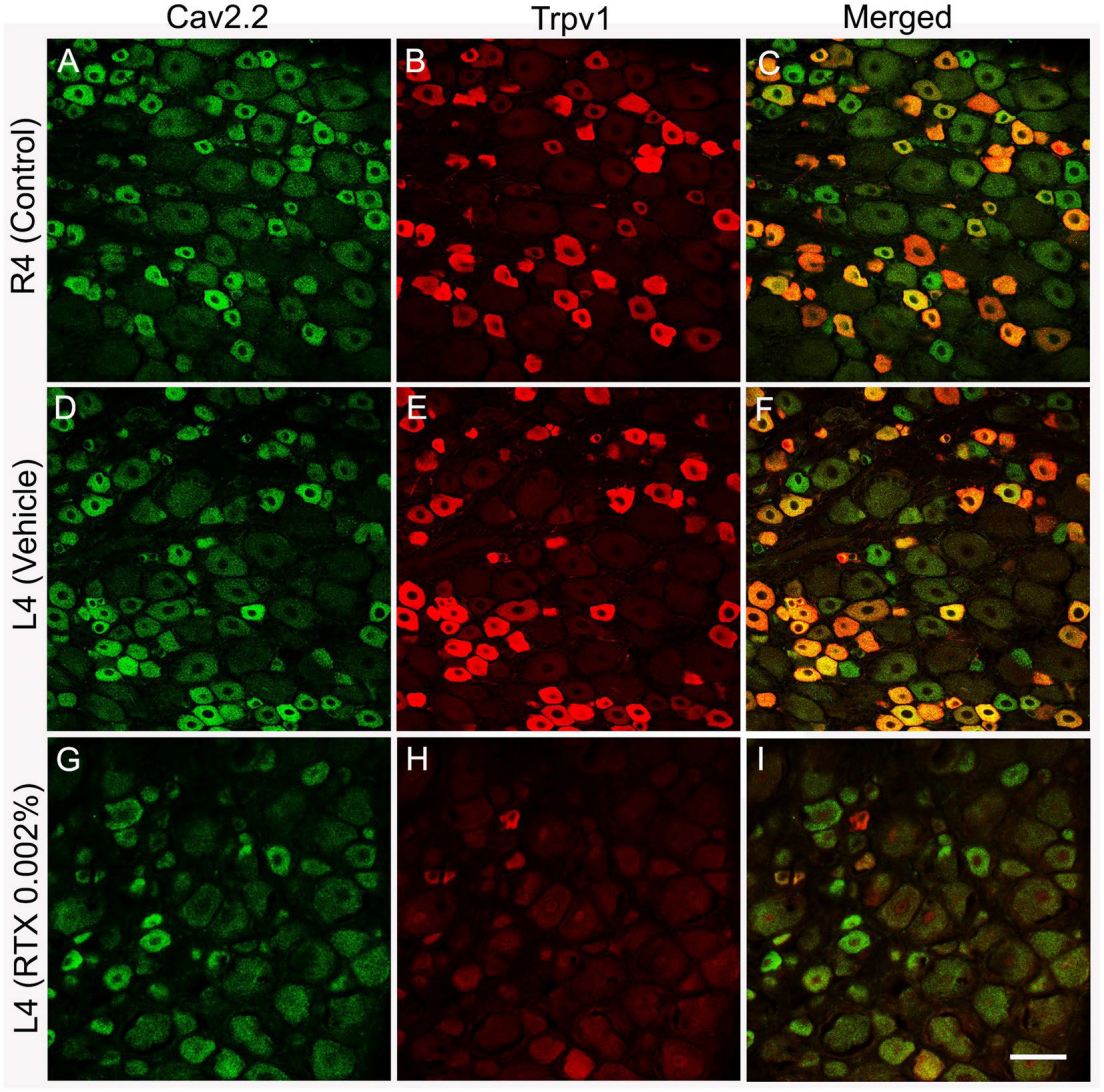
Double immunofluorescent labeling shows that both Cav2.2+ **(A,D)** and Trpv1 **(B,E)** profiles were mainly identified in small-sized neurons in the L4 control DRG with a considerable colocalization **(C,F)**. RTX application on the L4 nerve caused a significant reduction in the number of Cav2.2+ (**G**, **p < 0.01) and Trpv1+ (**H**, ***p < 0.001) neurons in the corresponding L4 DRG compared to the right control **(A,B)** and vehicle-treated L4 DRGs **(D,E)**. *n* = 3–4, scale bar = 50 μm.

**Figure 5:**
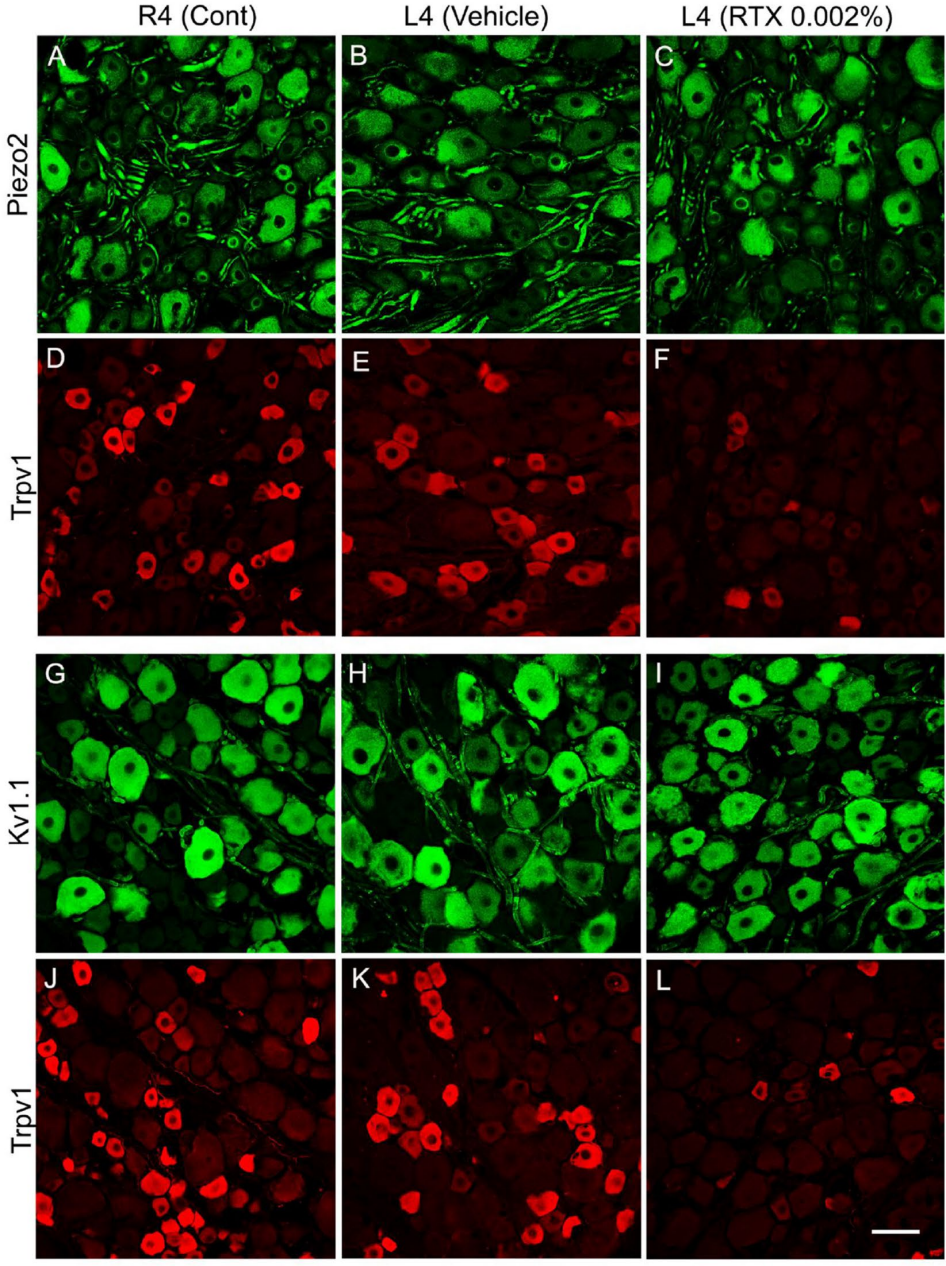
Double immunofluorescent labeling of either Piezo2 **(A–C)** and Trpv1 **(D–F)** or Kv1.1 **(G–I)** and Trpv1 **(J–L)** neurons in control right L4 DRG (R4 cont, **A,D,G,J**) and left L4 DRGs following 14 days of the perineural application of vehicle **(B,E,H,K)** or 0.002% RTX **(C,F,I,L)** on the left L4 nerve. Piezo2+ **(A–C)** and Kv1.1+ **(G–I)** immunoreactivities were mainly localized in large-sized neurons, while Trpv1+ neurons were mainly found in small-sized neurons (**D–F**, **J–L**) with negligible colocalization. RTX treatment showed a significant reduction in the number of Trpv1+ neurons (***p < 0.001) **(F,L)** but produced no effect on the Piezo2+ **(C)** or Kv1.1+ neurons **(L)** (*p* > 0.05) (I) in the L4 DRG compared to the right control and vehicle-treated L4 DRGs **(A,B,G,H)**. *n* = 3–4, scale bar = 50 μm.

Double and triple immunofluorescent staining revealed that Trpv1, Nav1.9, Kv4.3, and Cav2.2 immunoreactivities were mainly localized in numerous small-sized neurons of the control L4 DRG (Figures 1–5). Cross-sectional areas for Trpv1-, Nav1.9-, Kv4.3-, and Cav2.2-labeled neuron profiles ranged from 100 μm^2^–1,000 μm^2^ with a peak at 200 to 400 μm^2^. Only 2.6, 0.4, 2.5 and 3.6% of Trpv1-, Nav1.9-, Kv4.3-, and CaV2.2-labeled neuron profiles, respectively were larger than 800 μm^2^ (Figure 1). The percentage distribution of Trpv1-, Nav1.9-, Kv4.3-, and Cav2.2-labeled neurons was with an average of 36, 39, 44, and 45% of the total number of DRG neurons, respectively, (Tables 2A–C). Further quantitative analysis showed that 54% of Nav1.9+, 59% Cav2.2+, and 41% Kv4.3+ neurons were colocalized with Trpv1+ neurons in the control L4 DRG (Tables 2A–C; Figures 2–4). In comparison, the majority of Kv1.1- (91.1%), Piezo2- (81.2%) labeled neurons were mainly localized in large-sized neurons with cross-sectional areas of more than 800 μm^2^ (Figure 1) with negligible colocalization with Trpv1 (Figure 5; Tables 2A,D). Kir4.1 was localized in the satellite cells surrounding the DRG neurons with no colocalization with Trpv1 labeled neurons (Supplementary Figures S1, S2).

**Table 2:** The percentage distribution of nociceptive ion channels in the left L4 DRGs after 14 days of perineural application of RTX (0.002%) compared with vehicle-treated L4 and contralateral right (R4) DRGs.

*A* *n* = 4	TRPV1/ DAPI (%)	Nav1.9/ DAPI (%)	Kv1.1/ DAPI (%)	Nav1.9 colocalization with TRPV1 (%)	Kv1.1 colocalization with TRPV1 (%)
Vehicle L4	36.04 ± 4.96	38.20 ± 6.14	40.01 ± 1.27	54.32 ± 2.0	2.26 ± 0.7
RTX L4	3.50 ± 1.02***	15.81 ± 3.92*	44.95 ± 2.02	7.95 ± 1.65	1.11 ± 0.8
Non-RTX R4	35.96 ± 3.46	38.64 ± 5.22	39.2 ± 1.46	55.7 ± 3.79	2.3 ± 0.47

*B* *n* = 3–4	TRPV1/ DAPI (%)	Kv4.3/ DAPI (%)	Kv4.3 colocalization with TRPV1 (%)
Vehicle L4	31.39 ± 1.68	44.91 ± 0.44	41.38 ± 1.30
RTX L4	1.50 ± 0.49***	24.50 ± 4.7*	3.69 ± 1.72
Non-RTX R4	29.88 ± 1.91	42.19 ± 3.77	44.09 ± 1.43
*C* *n* = 3–4	TRPV1/ DAPI (%)	Cav2.2/ DAPI (%)	Cav2.2 colocalization with TRPV1 (%)
Vehicle L4	41.41 ± 0.76	45.5 ± 1.66	59.36 ± 2.9
RTX L4	4.7 ± 0.57***	34.1 ± 0.75**	7.83 ± 1.22
Non-RTX R4	38.21 ± 1.48	43.99 ± 1.28	62.90 ± 1.66
*D* *n* = 3	TRPV1/ DAPI (%)	Piezo2/ DAPI (%)	Piezo2 colocalization with TRPV1 (%)
Vehicle L4	30.13 ± 1.36	25.18 ± 0.84	4.35 ± 0.64
RTX L4	0.71 ± 0.52***	26.09 ± 1.16	0 ± 0
Non-RTX R4	35.51 ± 2.81	25.09 ± 1.14	5.60 ± 0.51

A significant decrease in the number of Trpv1, Nav1.9, Ca_V_2.2 and K_V_4.3 immunoreactive neurons was observed in the ipsilateral L4 DRG compared with left L4 vehicle-treated or contralateral control right L4 DRGs. No significant decrease was observed in the number of K_V_1.1 and Piezo2 immunoreactive neurons. No significant difference was observed in the number of labeled neurons of all above mentioned sensory markers between vehicle-injected L4 and control right L4 DRGs. The number of labeled neurons in all animals was counted in five sections per DRG and one-way ANOVA with Bonferroni *post hoc* test was used for the statistical analysis. ***p < 0.001; **p < 0.01; *p < 0.05 comparison for L4 RTX-treated vs. L4 vehicle-treated or control right L4 nerves.

### 3.2 Effects of perineural application of 0.002% RTX on L4 nerve on the expression of Trpv1, Nav1.9, Kv4.3, Cav2.2, Kv1.1, Piezo2, and Kir4.1 in L4 DRG

The number and the percentage of neurons expressing ion channels were calculated in the RTX-treated or vehicle-treated ipsilateral and contralateral L4 control DRGs. Quantitative analysis showed that the number of Trpv1+ neurons was markedly and significantly reduced (*p* < 0.001) in the L4 DRGs after RTX injection compared to vehicle-treated and right control L4 DRGs (Figures 2–5; Supplementary Figure S2; Tables 2A–D). Similarly, a significant reduction in the number of Nav1.9+ (*p* < 0.05), Kv4.3+ (*p* < 0.05) and Cav2.2+ (*p* < 0.01) neurons was also observed in the RTX-treated ipsilateral L4 DRGs compared to vehicle-treated L4 DRG (Figures 2–4; Tables 2A–C). In comparison, there was no significant difference in the number of Nav1.9+ (*p* > 0.05), Kv4.3+ (*p* > 0.05) and Cav2.2+ (*p* > 0.05) neurons in vehicle-treated L4 DRG compared to right control L4 DRG (Tables 2A–C).

Next, we investigated the effect of the perineural application of RTX 0.002% on the expression of other ion channels, Kv1.1, Piezo2, and Kir4.1, in the neurons of DRG which are not colocalized with Trpv1 labeled neurons. The results showed that there were no significant differences in the number of neurons that expressed Kv1.1 (*p* > 0.05) or Piezo2 (*p* > 0.05) in the DRGs treated with RTX and vehicle, as well as right control DRGs (Figure 5; Tables 2A,D). Our immunohistochemical data also did not show a significant effect on Kir4.1 expression in the satellite cells in the RTX-treated ipsilateral L4 DRG compared to vehicle-treated and right control DRGs (Supplementary Figures S1, S2).

### 3.3 Effects of perineural application of 0.002% RTX on the mRNA and protein expression of various ion channels in the DRG

**Figure 6:**
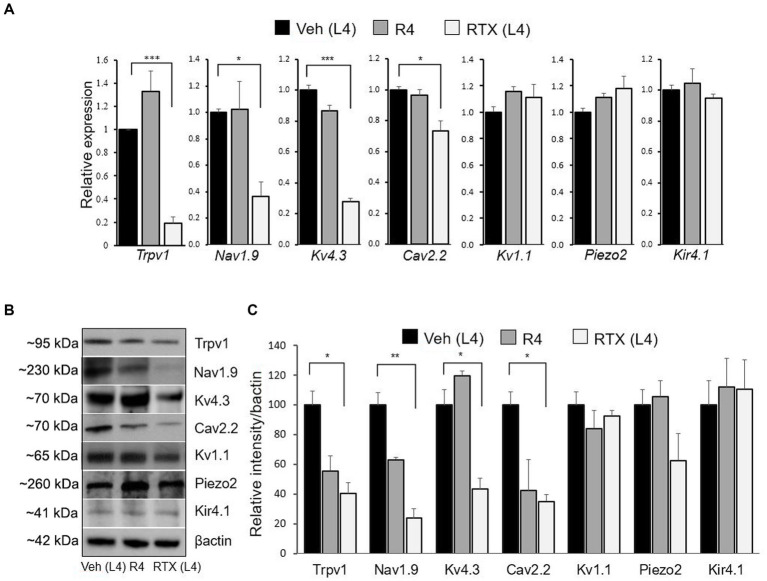
The mRNA and protein expression levels of the Trpv1, Nav1.9, Kv4.3, Cav2.2, Kv1.1, Piezo2, and Kir4.1 were evaluated after 14 days of 0.002% RTX or vehicle treatment on the left L4 nerve. A significant decrease in the mRNA level of Trpv1, Nav1.9, Kv4.3, and Cav2.2 was observed in the RTX-treated L4 DRG compared to vehicle-treated L4 and right control R4 DRGs, but no changes were found in the Kv1.1, Piezo2, and Kir4.1 **(A)**. Similarly, the protein expression level of Trpv1, Nav1.9, Kv4.3, and Cav2.2 was also significantly decreased in RTX-treated L4 DRG compared to vehicle-treated L4 and right control R4 DRGs. However, no change in the protein expression level was observed in Kv1.1, Piezo2, and Kir4.1 following RTX treatment **(B,C)**. ***p < 0.001; **p < 0.01; *p < 0.05. Two-way ANOVA (*n* = 3–5 per group).

To support our immunohistochemical data, we also performed qRT-PCR and western blotting to investigate the mRNA and protein expression of Trpv1, Nav1.9, Kv4.3, Cav2.2, Kv1.1, Piezo2, and Kir4.1 ion channels in the DRGs. The results of qRT-PCR showed a significant (*p* < 0.05; *p* < 0.001) decrease in the mRNA expression of Nav1.9, Kv4.3, Cav2.2, and Trpv1 in the RTX-treated ipsilateral L4 DRG compared to vehicle-treated L4 and right control R4 DRGs (Figure 6A). However, no significant differences were observed in Kv1.1, Piezo2, and Kir4.1 mRNA expression between RTX-treated ipsilateral L4 DRG and vehicle-treated L4 as well as right control L4 (R4) DRGs (Figure 6A). In addition, we also analyzed the protein expression levels of Trpv1, Nav1.9, Kv4.3, Cav2.2, Kv1.1, Piezo2, and Kir4.1 ion channels in the RTX-treated ipsilateral and contralateral right control as well as vehicle-injected ipsilateral L4 DRGs. We observed significantly decreased expression (*p* < 0.05; *p* < 0.01) of Trpv1, Nav1.9, Kv4.3, and Cav2.2 in the RTX-injected ipsilateral L4 DRG compared to the vehicle-injected and right control L4 DRGs (Figures 6B,C). However, no significant difference was observed in the expression level of Kv1.1, Piezo2, and Kir4.1 in the RTX-treated L4 DRG compared to the vehicle-treated and right control DRGs (Figures 6B,C).

### 3.4 Effects of perineural application of 0.002% RTX on the immunoreactivity of Trpv1, IB4, NF200, and Kv1.1 in the treated L4 nerve

**Figure 7:**
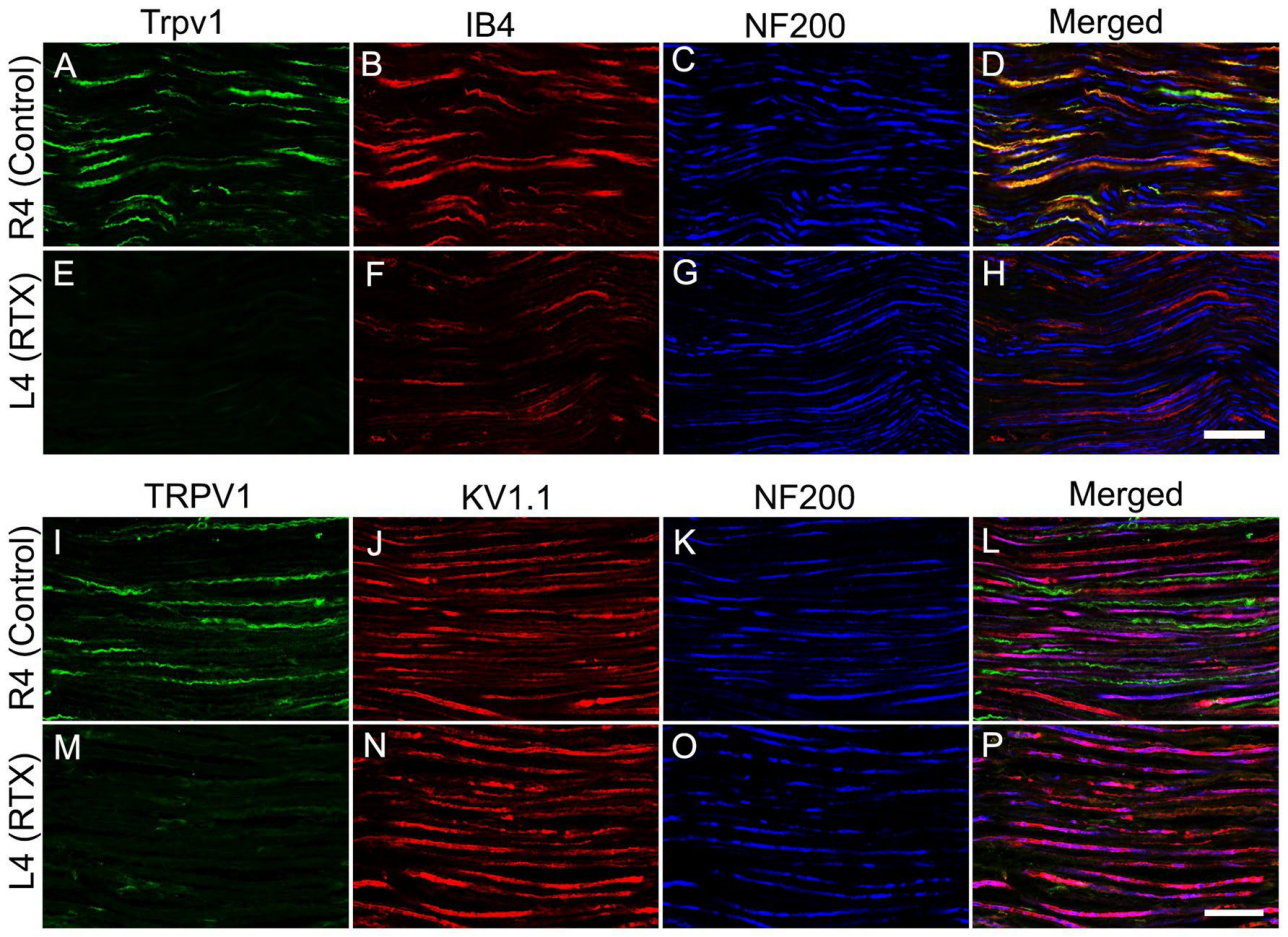
Confocal images of triple immunofluorescent labeling for Trpv1, IB4, and NF200 **(A-D)** and Trpv1, Kv1.1, and NF200 **(I-L)** in control right L4 nerve and following the perineural application of 0.002% RTX on the left L4 nerve **(E–H, M–P)**. A distinct colocalization of Trpv1 with IB4 and an absence of colocalization with Kv1.1 and NF200 in the normal L4 nerve. Furthermore, in the RTX-treated ipsilateral L4 nerve, we observed significant reductions in both Trpv1 and IB4 immunoreactivities (markers for small-sized sensory neurons), while the expression level of Kv1.1 and NF200 (markers for large-sized neurons) remained largely unchanged compared to the unaffected contralateral control L4 nerve. Scale bar = 25 μm.

Lastly, we investigated the immunofluorescent expression of Trpv1, IB4 binding (as two markers of small-sized neurons), Kv1.1 and NF200 (as two markers of lager-sized neurons) in the RTX-treated and right control L4 nerves to support our previous findings. We observed a clear colocalization of Trpv1 with IB4 and a lack of colocalization with Kv1.1 and NF200 in the control L4 nerve (Figures 7A–D,I). In addition, we observed marked downregulation of Trpv1 and IB4 immunoreactivities (Figures 7E,F,M) with no clear effect on the expression level of NF200 (Figures 7G,O) and Kv1.1 (Figure 7N) in the RTX-treated ipsilateral L4 nerve compared to the contralateral right control L4 nerve (Figures 7A–D,I). These findings showed that RTX selectively affected unmyelinated and possibly thin myelinated nerve fibers, which contained Trpv1 and IB4, while no effect on myelinated nerve fibers, which expressed Kv1.1 and NF200.

### 3.5 Effects of perineural application of 0.002% RTX on c-fos activation in the spinal cord in response to heat stimuli

**Figure 8:**
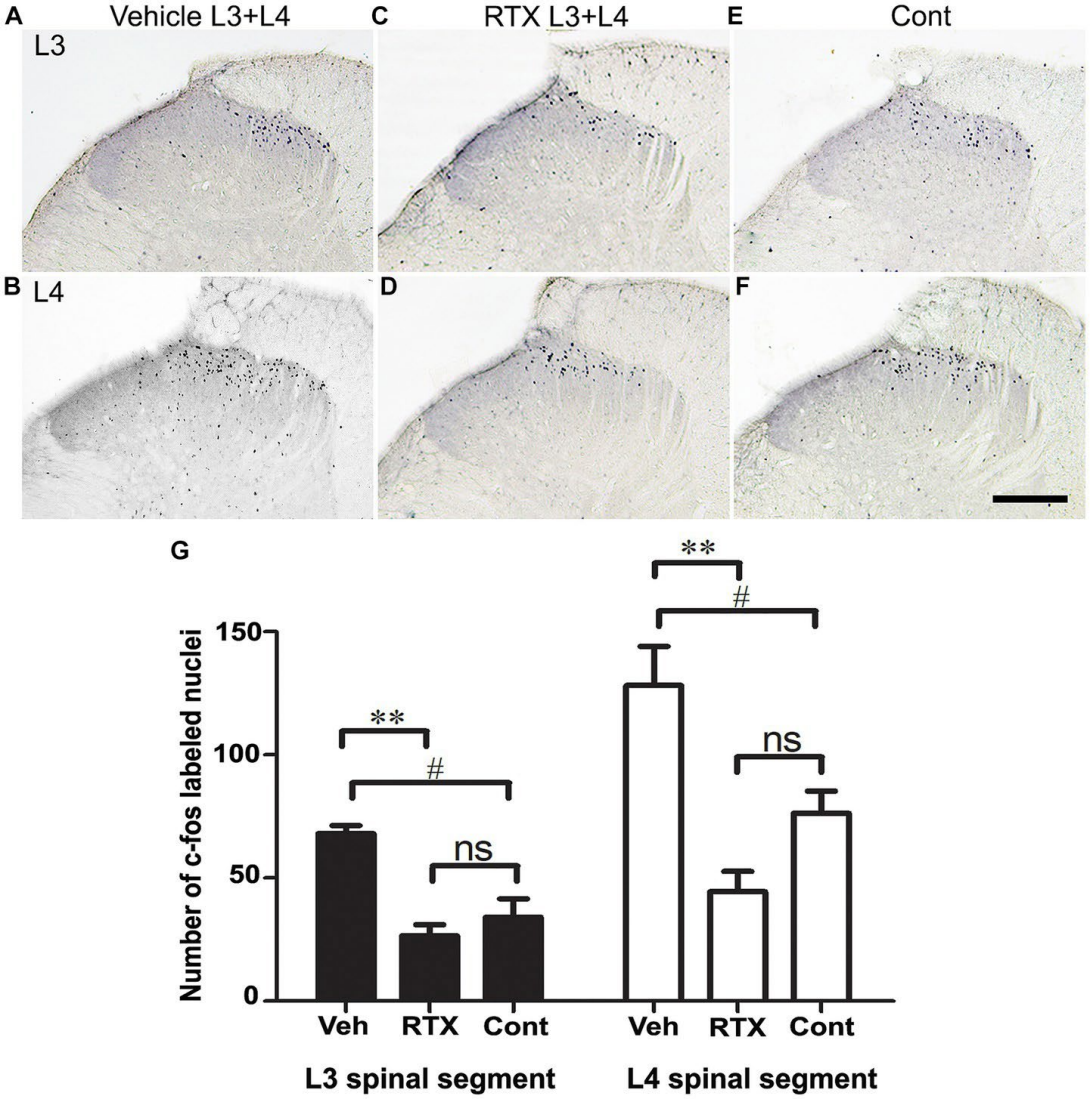
Images in **(A–F)** show c-fos expression in sections of L3 and L4 spinal segments 90 min after heat stimulation of both hind paws of rats whose left L3 and L4 nerves were perineurally treated with either 0.002% RTX or vehicle and L5 nerve was injured 21 days earlier. A profound expression of c-fos was observed in the medial half of the superficial laminae (I and II) of the dorsal horn of L3 **(A)** and L4 **(B)** spinal segments of animals that were treated with a vehicle compared with control side **(E,F)**. In contrast, animals treated with RTX showed a significant reduction in c-fos expression in the dorsal horn of L3 **(C)** and L4 **(D)** spinal segments. The right control sides of L3 **(E)** and L4 **(F)** showed the level of c-fos expression in the dorsal horn following heat stimulation. (Scale bar = 200 μm). Data analysis in hitograms **(G)** showed a significant (#*p* < 0.05; One-way ANOVA) increase in the number of c-fos labeled nuclei in the left sides of the vehicle-treated rats compared with the control right sides of L3 and L4 spinal segments. This indicates a thermal hypersensitivity in the vehicle-treated rats due to L5 nerve injury. In comparison, there was a significant reduction (**p < 0.01; One-way ANOVA) in the number of c-fos labeled nuclei in the L3 and L4 spinal segments of RTX-treated rats compared to the vehicle-treated rats. This indicates an analgesic effect and a suppression of the thermal hypersensitivity in the hind paw in response to RTX treatment. However, no significant difference in the number of c-fos labeled nuclei was observed in the RTX-treated rats compared with the control right sides of the L3 and L4 segments. (*n* = 3).

In this experiment, we used c-fos as a neuronal activity marker (Dragunow and Faull 1989; Bullitt, 1990; Shehab et al., 2014). In normal naïve rats, c-fos is not detectable in sections of the spinal cord, and only very few labeled nuclei might be observed in the dorsal horn. In comparison, painful stimuli applied to the hind paws would cause the activation of small-diameter cutaneous sensory afferents, resulting in rapid induction of c-fos in many neuronal nuclei in the medial half of the dorsal horn. The expression of c-fos in the neurons in the medial half of the dorsal horn (Figures 8A–F) is consistent with somatotopically arranged termination of the primary afferents of the foot (Hunt et al., 1987; Todd et al., 1994; Shehab et al., 2022). To assess the effect of RTX on the c-fos activation in the dorsal horn of the spinal cord, the left L3 and L4 spinal nerves were treated with 0.002% RTX or vehicle perineurally, and the L5 nerve was ligated and cut. After 3 weeks (a period when the effects of RTX would have taken place and neuropathic pain would have been established), both hind paws of the animals were immersed in the hot water bath at 50°C temperature for 2 min. After 90 min of heat treatment, rats were perfused, and c-fos immunohistochemistry was performed in L3 and L4 spinal segments. The contralateral right side dorsal horn of the spinal cord of RTX-treated rats was considered as the control. The results showed the number of c-fos labeled nuclei in the superficial layer of the dorsal horn (laminae I-II) was significantly increased in both L3 (*p* < 0.05) and L4 (*p* < 0.05) spinal segments in the injury side of vehicle-treated rats than the right contralateral control side (Figures 8). This indicated the presence of thermal hypersensitivity and hyperalgesia in the ipsilateral hind paw due to L5 nerve injury. In comparison, quantitative data showed a significant decrease (*p* < 0.01) in the number of c-fos labeled nuclei in the dorsal horn of both L3 and L4 spinal segments of RTX-treated rats compared to vehicle-treated rats (Figure 8). Furthermore, although RTX treatment produced a smaller number of c-fos labeled nuclei in the dorsal horn of both L3 and L4 spinal segments compared to the contralateral right control side, no significant difference was found (Figure 8). The results of c-fos experiment are consistent with the behavior data which showed that RTX treatment markedly reduced the thermal hypersensitivity and the response to heat stimuli applied to the hind paw returned to a basal level (see next).

### 3.6 Effects of perineural application of RTX to L3 and L4 nerves on the prevention of thermal and mechanical hypersensitivity caused by L5 nerve injury

**Figure 9:**
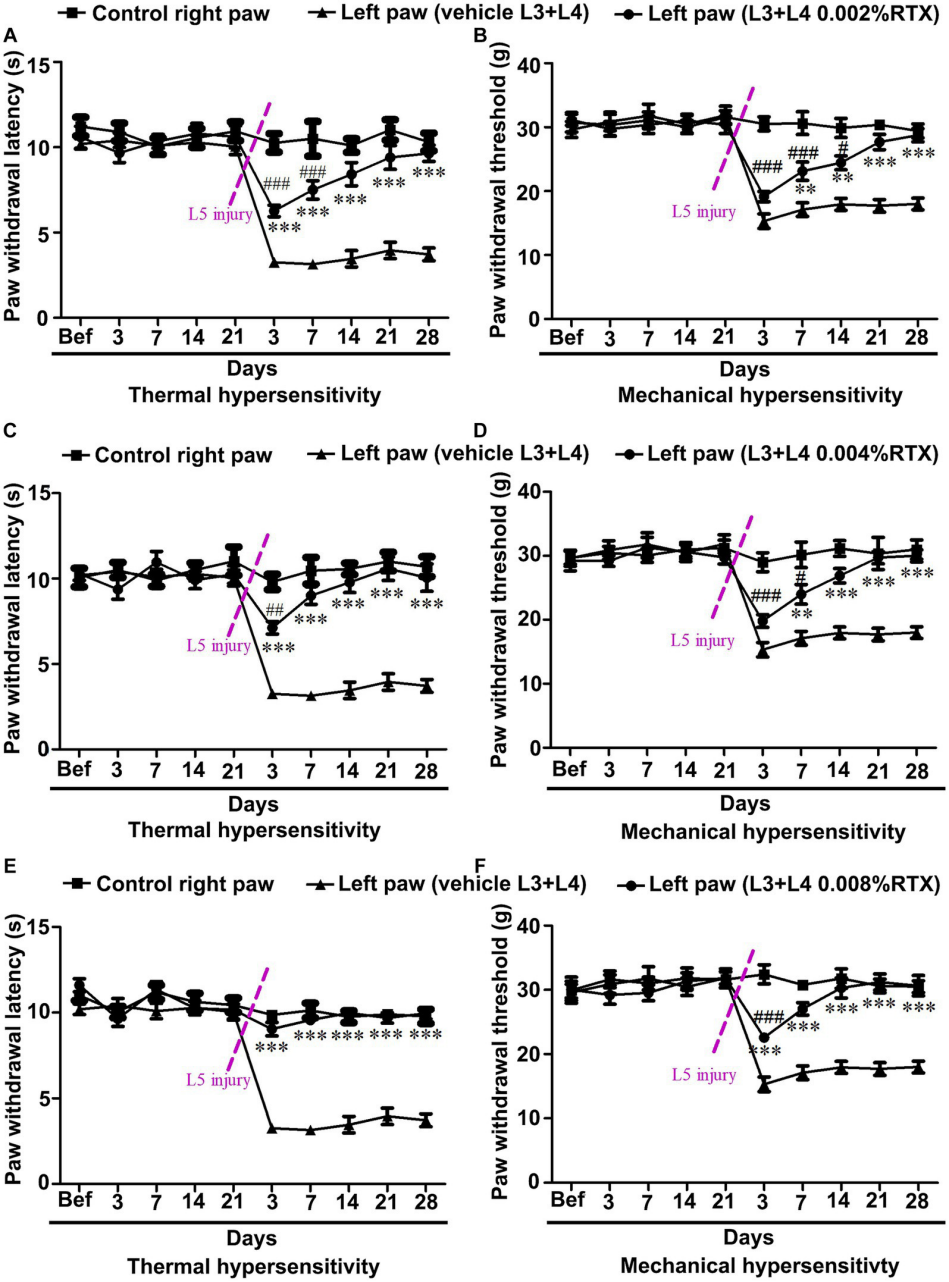
Dose-dependent effect of perineural application of RTX (0.002, 0.004, and 0.008%) on preventing the development of L5 nerve injury-induced thermal and mechanical hypersensitivity. Pre-treatment of left L3 and L4 nerves with 0.002, 0.004, and 0.008% RTX did not show any significant difference in thermal and mechanical paw withdrawal latencies of RTX-treated rats compared to vehicle and right-side control rats following 3–21 days of treatment **(A–F)**. In these animals, the left L5 nerve injury was carried out to induce neuropathic pain after 21 days of RTX/vehicle treatment. Animals pre-treated with 0.002% **(A)** and 0.004% **(B)** RTX showed insignificant left paw thermal withdrawal latency compared to right control paw withdrawal latency 14 and 7 days after L5 nerve injury, respectively and continued till 28 days. In comparison, animals pre-treated with a higher dose of 0.008% RTX showed left paw thermal withdrawal latency equivalent (reached to baseline values) to right control paw withdrawal latency after 3 days and continued to 28 days after L5 nerve injury. The mechanical left paw withdrawal latency was also significantly increased in animals pre-treated with RTX compared to the left paw withdrawal latency of vehicle-treated rats at 7–28 days (0.002, and 0.004%) and 3–28 days (0.008%) after L5 nerve injury **(D-F)**. The left paw mechanical withdrawal latencies of animals pre-treated with RTX doses 0.002, 0.004, and 0.008% reached the baseline values at 21, 14, and 7 days after left L5 nerve injury, respectively, and this effect of RTX continued till the end point of experiments at 28 days. [(*RTX vs. vehicle; #RTX vs. right control) **p < 0.01; ***p < 0.001; #*p* < 0.05; ##*p* < 0.01; ###*p* < 0.001; *n* = 7 each group].

In the current study, we aimed to prevent thermal and mechanical hypersensitivity at an early time. To do that, left L3 and L4 nerves were pre-treated by perineural application of RTX with an increasing concentration (0.002, 0.004, and 0.008%) or vehicle. The results of all three doses of RTX (0.002, 0.004 and 0.008%) showed no significant changes in the thermal and mechanical left paw withdrawal latencies from 3 to 21 days post-treatment compared to the right control and vehicle-treated left paws (Figures 9A–F). After 21 days of RTX application, the rats underwent left L5 nerve injury, and they were again tested for both thermal and mechanical paw withdrawal latencies from 3 to 28 days post L5 nerve injury. The results showed a significant (*p* < 0.001) increase in thermal paw withdrawal latencies in rats treated with all three doses of RTX compared to vehicle-treated control rats from 3–28 days post L5 nerve injury (Figures 9A–C). However, rats were still significantly (*p* < 0.001; *p* < 0.01) hypersensitive/hyperalgesic at particular time points (0.002% at 3 and 7 days post-injury and 0.004% at 3 days post-injury) compared to right control paw withdrawal latencies (Figures 9A,B). Interestingly, the higher dose of RTX (0.008%) completely prevented the thermal pain as there was no significant difference observed in the thermal paw withdrawal latency of the RTX-treated left paw and right control paw after 3 days of L5 nerve injury (Figure 9C). Furthermore, this robust and complete analgesic effect of 0.008% of RTX continued 3–28 days after L5 nerve injury.

A similar effect of RTX on mechanical hypersensitivity was also observed. The mechanical left paw withdrawal threshold was significantly (*p* < 0.001; *p* < 0.01) increased in rats treated with 0.002 and 0.004% RTX compared to the left paw withdrawal threshold of vehicle-treated rats after 7–28 days post L5 nerve injury (Figures 9D,E). In comparison, the higher dose of RTX (0.008%) significantly (*p* < 0.001) increased the mechanical paw withdrawal latency one-time point earlier, i.e., 3 days after L5 nerve injury (Figure 9F) and it reached the basal level after 7 days. The current study showed that all doses of RTX significantly prevented the mechanical hypersensitivity caused by L5 nerve injury, with maximum effects observed in rats treated with RTX 0.008%. Interestingly, rats pre-treated with 0.002% RTX displayed insignificant mechanical paw withdrawal latencies compared to the right control after 21–28 days of L5 nerve injury. In comparison, however, 0.004 and 0.008% RTX doses showed this effect after 14–28 and 7–28 days, respectively. The behavioral data of the current study showed that the pre-treatment of left L3 and L4 nerves with 0.008% RTX completely prevented thermal hypersensitivity induced by L5 nerve injury and attained the baseline level of thermal paw withdrawal latency just after 3 days of injury but its effect on achieving the baseline values of mechanical paw withdrawal latency started a week later following L5 nerve injury.

### 3.7 Effects of perineural application of 0.008% RTX on the ultrastructural alterations in unmyelinated axons of L4 nerve and DRGs neurons

**Figure 10:**
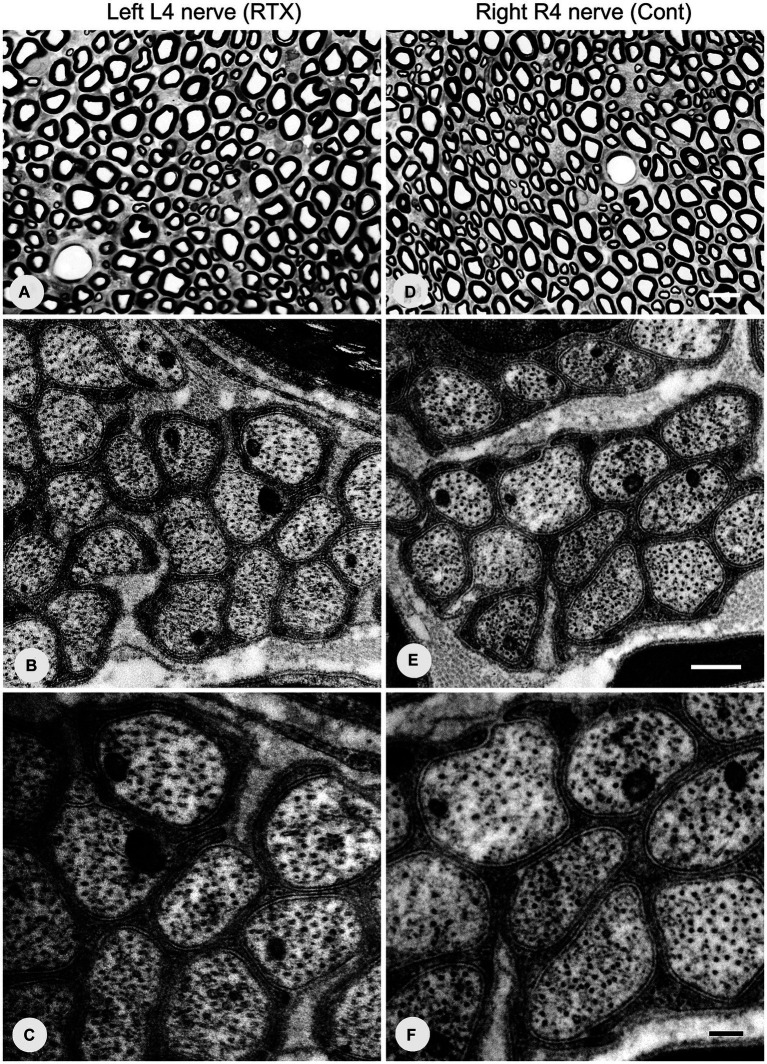
No morphological or degenerative changes were observed in the left and right control L4 nerves after the perineural application of 0.008% RTX on the left L4 nerve. Semi-thin sections of the left L4 nerve **(A)** and right L4 (R4) **(D)** nerves were stained with toluidine blue (1%). Ultrastructural observations at magnifications of 26,500× **(B,E)** and 43,000× **(C,F)** revealed a group of unmyelinated axons of the L4 nerves on the RTX-treated side with normal morphology. Scale bar in **(A,D)** = 10 μm; **(B,E)** = 500 nm; and **(C,F)** = 200 nm.

**Figure 11:**
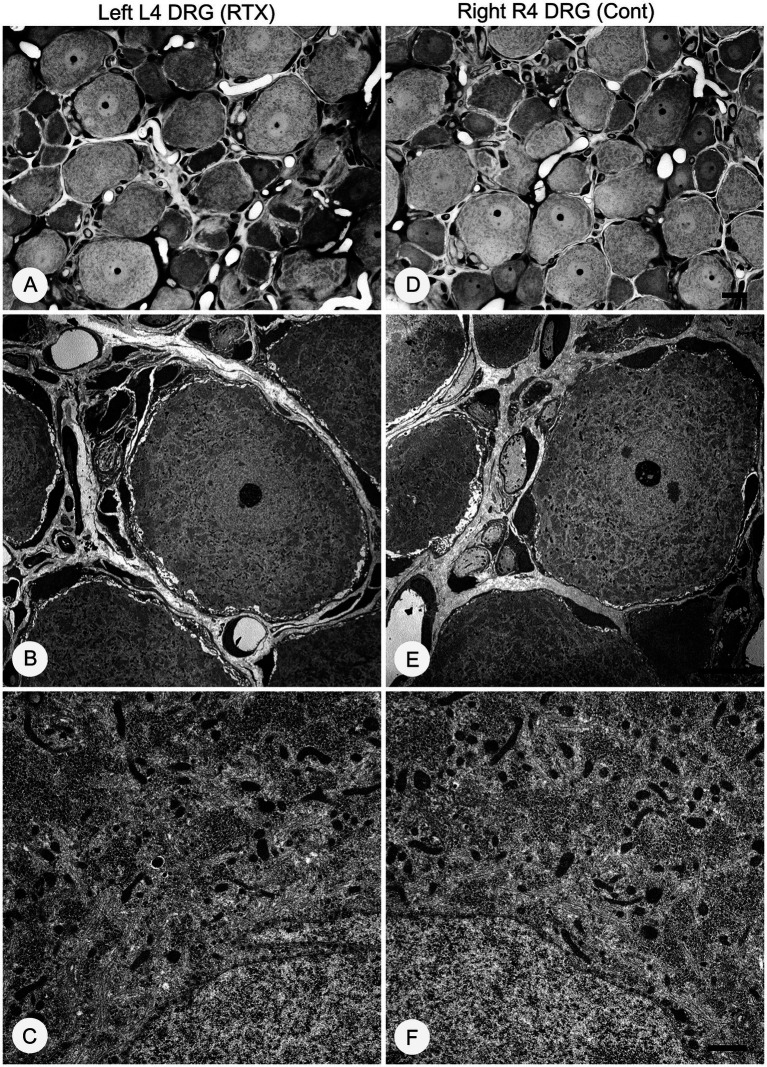
Semi-thin **(A,D)** and ultra-thin **(B,C,E,F)** sections showing no morphological changes in the left L4 DRG **(A–C)** compared with the right control side **(D–F)** 14 days after 0.008% RTX application on the L4 nerve. Characteristic morphology of neurons was observed in semi-thin sections of RTX-treated left and right control DRGs stained with toluidine blue **(A,B)**. Electron micrographs at low (1,250×) and high (9,900×) magnifications showed the normal appearance of small- and medium-sized neurons. These micrographs also reveal normal architecture of mitochondria and endoplasmic reticula with intact nuclear membrane and other subcellular organelles in DRG neurons on the treated side **(B–C)** compared to right control DRG neurons **(E,F)**. Scale bar **(D)** = 20 μm; **(E)** = 10 μm; and **(F)** = 1 μm.

Having established the best dose of RTX (0.008%) that produced complete prevention of thermal and mechanical hypersensitivity, we next investigated whether this analgesic effect of RTX treatment was due to degeneration in the unmyelinated axons and DRG neurons supplying the hind paw. To achieve that, 0.008% of RTX was applied to the L4 nerve, and 14 days later, the L4 nerve and its corresponding DRG were examined at both light and EM levels. Semi-thin sections (1.5 μm thickness) of L4 nerves were stained with toluidine blue (1%) to reveal the overall normal architectural morphology under light microscopy (Figures 10A,D). A critical examination at higher magnifications of up to 43,000× was carried out using TEM, and the results showed no evidence of any morphological alterations such as swelling, disintegration, abnormal deposits, destruction, or degeneration of cellular organelles in the unmyelinated axons (Figures 10B,C,E,F). In addition, a series of 20 micrographs per cross-section were acquired (two sections/animal) at a magnification of 16,500×. Images were used to determine the size of unmyelinated axons in L4 nerves. The quantitative analysis showed that there was no significant difference (*p* > 0.05) in the sizes of unmyelinated axons between the RTX-treated left nerve and right control L4 nerves and between treated-left and vehicle-treated left L4 nerves (Table 3). Likewise, semi-thin and ultrathin sections of the DRGs were also examined for any morphological abnormalities, such as degeneration of cellular organelles and membranous disintegrations in small- and medium-sized neurons. As shown in Figure 11 we observed no morphological differences between neurons of the left RTX-treated DRGs and the right control DRGs in terms of signs of damage or degeneration, and the morphology of cellular organelles, such as mitochondria, Golgi complex, and endoplasmic reticulum, as well as plasma and nuclear membranes, was normal.

**Table 3:** TEM examination showing that the perineural application of RTX (0.008%) on the left L4 nerve for a period of 14 days did not result in any significant changes in the size of the unmyelinated axons in the ipsilateral left L4 nerves, when compared to the left vehicle-treated and right control L4 nerves (*n* = 3).

Samples 0.008% RTX	No. images (16,500×) from two cross-sections per animal	The perimeter of unmyelinated axons (μm) ± SEM	No. of degenerated axons observed
Left RTX-treated L4 nerve	40	2.36 ± 0.27	0
Left vehicle-treated L4 nerve	40	2.36 ± 0.09	0
Right R4 nerve	40	2.51 ± 0.07	0

## 4 Discussion

Treatments of human neuropathic pain are considered to be effective when they result in a 30–50% reduction in pain measurements (Attal et al., 2011). However, our primary objective was to achieve a complete blockade of neuropathic pain. The innovative aspect of our experimental approach lies in treating and preventing nerve injury-induced neuropathic pain through the direct treatment of adjacent uninjured nerves. Our study demonstrates that perineural application of RTX to the uninjured L3 and L4 nerves could completely prevent the development of thermal and mechanical hypersensitivity caused by L5 nerve injury in a dose-dependent manner. Although both 0.002 and 0.004% concentrations of RTX produced significant analgesic effects, a concentration of 0.008% of RTX was necessary to prevent the onset of thermal and mechanical hypersensitivities completely.

### 4.1 Comparison with other studies

Our previous study (Javed et al., 2020) demonstrated that perineural application of 0.002% RTX to the uninjured L3 and L4 abolished neuropathic pain manifestations following L5 nerve injury. In that study, the application of RTX was performed at the same time as the L5 nerve injury. The results showed a significant reduction in thermal hypersensitivity that began after 7 days, and paw withdrawal latencies in response to thermal stimuli returned to baseline levels after 3 weeks. Similarly, mechanical hypersensitivity was alleviated after 4 weeks. In the current study, we employed different doses of RTX on the L3 and L4 nerves 3 weeks prior to L5 nerve injury to prevent the development of neuropathic pain. Complete prevention of thermal hypersensitivity was observed during the initial testing conducted after 3 days, and complete prevention of mechanical hypersensitivity was observed after 7 days post-surgery.

In a different approach to evaluate the effects of local RTX application, we recently investigated the impact of intraplantar injection of RTX, rather than perineural application, on developing and preventing nerve injury-induced neuropathic pain in rats (Javed et al., 2022). The study’s main findings revealed that intraplantar injection of RTX also alleviated and prevented nerve injury-induced thermal and mechanical hypersensitivity. However, there are several advantages to utilizing perineural application over intraplantar injection, particularly when attempting to translate this therapeutic approach for treating and preventing neuropathic pain in humans. First, rats showed paw flicking and guarding shortly after recovery from general anesthesia and exhibited foot edema following intraplantar injection (Javed et al., 2022). These observations were not witnessed after the perineural application of RTX. Second, the perineural application of RTX on two to three lumbar nerves in humans would affect a broader area of the skin of the lower limb. Third, the perineural application requires a significantly smaller volume of RTX compared to multiple cutaneous injections.

### 4.2 Local application of RTX does not cause nerve degeneration.

Initial research suggested that the analgesic effect achieved through the peripheral application of capsaicin and RTX could be associated with nerve degeneration (Jancsó and Lawson, 1990; Pini et al., 1990). However, our recent investigation (Javed et al., 2020) and studies conducted by other researchers (Ainsworth et al., 1981; Lynn and Shakhanbeh, 1988) have provided compelling evidence for the absence of degeneration in C-fibers in both control and treated nerves. Furthermore, no detectable ultrastructural changes were observed in the treated L4 nerve and DRG when RTX was applied perineurally at concentrations 0.002% (Javed et al., 2020) or 0.008% (current study). These findings are consistent with prior research demonstrating that the peripheral application of RTX does not cause any significant damage to unmyelinated nerve fibers (Kissin et al., 2007; Oszlács et al., 2015). In summary, based on the existing evidence, it can be concluded that alleviating and preventing neuropathic pain resulting from nerve injury through local application of RTX to adjacent uninjured nerves cannot be attributed to nerve degeneration.

### 4.3 Effects of perineural application of RTX to intact peripheral nerves on normal pain behavior

The plantar skin of the rat’s hind paw is supplied by L3-L5 nerves (Takahashi et al., 1994, 2002; Javed et al., 2020). Since uninjured nerves play a role in hyperalgesia and allodynia in neuropathic pain induced by peripheral nerve injury, both our previous study (Javed et al., 2020) and the work of other investigators (Jang et al., 2007) have demonstrated that chemical inactivation of L4 nerve with RTX or capsaicin leads to a significant attenuation in both thermal and mechanical hypersensitivity induced by L5 nerve injury. Nonetheless, to achieve a comprehensive alleviation of neuropathic pain symptoms, perineural application of RTX on both L3 and L4 nerves was required (Javed et al., 2020).

Similarly, in this study, when RTX was perineurally applied to the L3 and L4 nerves three weeks before the L5 nerve injury, it effectively prevented the manifestations of neuropathic pain in a dose-dependent manner. However, in control rats without L5 nerve injury, this treatment did not produce significant differences in their responses to thermal and mechanical stimuli. Similarly, another study demonstrated that the application of capsaicin solely on the L4 nerve did not affect the mechanical sensation (Figure 5) in Jang et al. (2007). Importantly, our previous investigation in which RTX was applied to all three nerves (L3-L5) that supply the entire plantar skin revealed no significant differences in withdrawal latency compared to the untreated hind paw. These findings indicate normal responses to thermal and mechanical stimuli from 3 to 28 days (Javed et al., 2020). Furthermore, other studies have shown that the perineural application of RTX blocks inflammatory hypersensitivity while minimally affecting normal thermal and mechanical sensations (Neubert et al., 2008). To support this, mice lacking Trpv1 exhibited normal responses to acute noxious stimuli but were unable to develop carrageenan-induced thermal hyperalgesia (Davis et al., 2000; Woodbury et al., 2004; Kim et al., 2012). These mice also showed minimal inflammatory thermal hyperalgesia and only exhibited modest behavioral responses to high radiant heat or noxious temperatures above 50–52°C (Caterina et al., 2000; Marics et al., 2014). This lack of effect against normal sensation indicates that the role of Trpv1 may be more prominent in the context of chronic pain states than the perception of acute painful stimuli (Woodbury et al., 2004; Javed et al., 2020). Additionally, it highlights the potential dissociation between the transmission of normal physiological pain and the abnormal hyperalgesia observed in neuropathic pain. Therapeutically, exploiting this dissociation may hold promise in effectively treating chronic pathological neuropathic pain.

### 4.4 Mechanisms of action perineural application of RTX

Understanding the mechanisms underlying the therapeutic potential of capsaicin and its analogue RTX in the management of neuropathic pain is of great importance. No detectable ultrastructural changes in the treated nerves and L4 DRG indicate that the prevention of the development of nerve injury-induced neuropathic pain following the treatment with 0.008% of RTX was not due to nerve degeneration. RTX primarily acts by activating Trpv1, which is predominantly expressed in small-sized sensory neurons in the DRG and unmyelinated peripheral nerves (Caterina et al., 1999; Ichikawa and Sugimoto, 2001; Hironaka et al., 2014; Brown, 2016; Javed et al., 2020). Activation of Trpv1 by RTX initiates neuroplastic changes in nociceptive neurons, leading to the downregulation of Trpv1 itself (Caterina and Julius, 2001; Karai et al., 2004; Premkumar and Sikand, 2008; Szigeti et al., 2012). This downregulation of Trpv1 following the application of perineural RTX may account for the attenuation and prevention of thermal hypersensitivity and hyperalgesia (Javed et al., 2020). However, the ability of RTX treatment to reduce mechanical hyperalgesia may be attributed to the downregulation of other nociceptive transmitters/receptors that coexist with Trpv1 neurons in the DRG. In particular, our study showed that RTX-induced down-regulation extended to other nociceptive ion channels, including Nav1.9, Kv4.3, and Cav2.2, within DRG neurons (this study). All these ion channels are expressed preferentially in small-sized neurons in DRG neurons (Huang et al., 2005; Wang et al., 2005; Bennett et al., 2019; Pitake et al., 2019; Kanda et al., 2021); with a significant colocalization with Trpv1 (this study), implicating their involvement in the perception, transmission, and modulation of pain signals. Furthermore, these ion channels play crucial roles in developing and maintaining nerve injury-induced neuropathic pain (Rasband et al., 2001; Vit et al., 2008; Dib-Hajj et al., 2015; Yang et al., 2018; Bennett et al., 2019; Zhang et al., 2019; Smith, 2020). In addition, local application of RTX also resulted in the downregulation of other well-known nociceptive neurotransmitters, which are also colocalized with Trpv1 in DRG neurons, such as calcitonin gene-related peptide (CGRP), substance P (SP), somatostatin, and isolectin B4 (IB4) binding (Jessell et al., 1978; Gamse, 1982; Gibson et al., 1982; Anand and Bley, 2011; Szigeti et al., 2012; Javed et al., 2020) and this study. Therefore, it is highly likely that numerous other nociceptive neurotransmitters, their receptors, and ion channels, which are found in small-sized neurons and coexist with Trpv1 in the DRG will be affected by RTX treatment. In comparison, RTX treatment had no impact on the expression of other ion channels, such as Kv1.1 and Piezo2, which are found in the large-sized neurons in the DRG and are not colocalized with the neurons expressing Trpv1 as seen in our study. Similarly, no effects on the expression of the Kir4.1 channel, which is localized in the satellite cells of the DRG, were observed. Consequently, our findings illustrate that RTX exerts a common denominator mechanism of action, leading to substantial down-regulation of multiple nociceptive mediators that are mainly found in Trpv1-containing neurons. The downregulation of multiple nociceptive mediators would ultimately lead to a reduction in pain transmission, thereby resulting in the alleviation and prevention of pain hypersensitivity, hyperalgesia, and allodynia.

### 4.5 Treatment of nerve injury-induced neuropathic pain in humans

**Figure 12:**
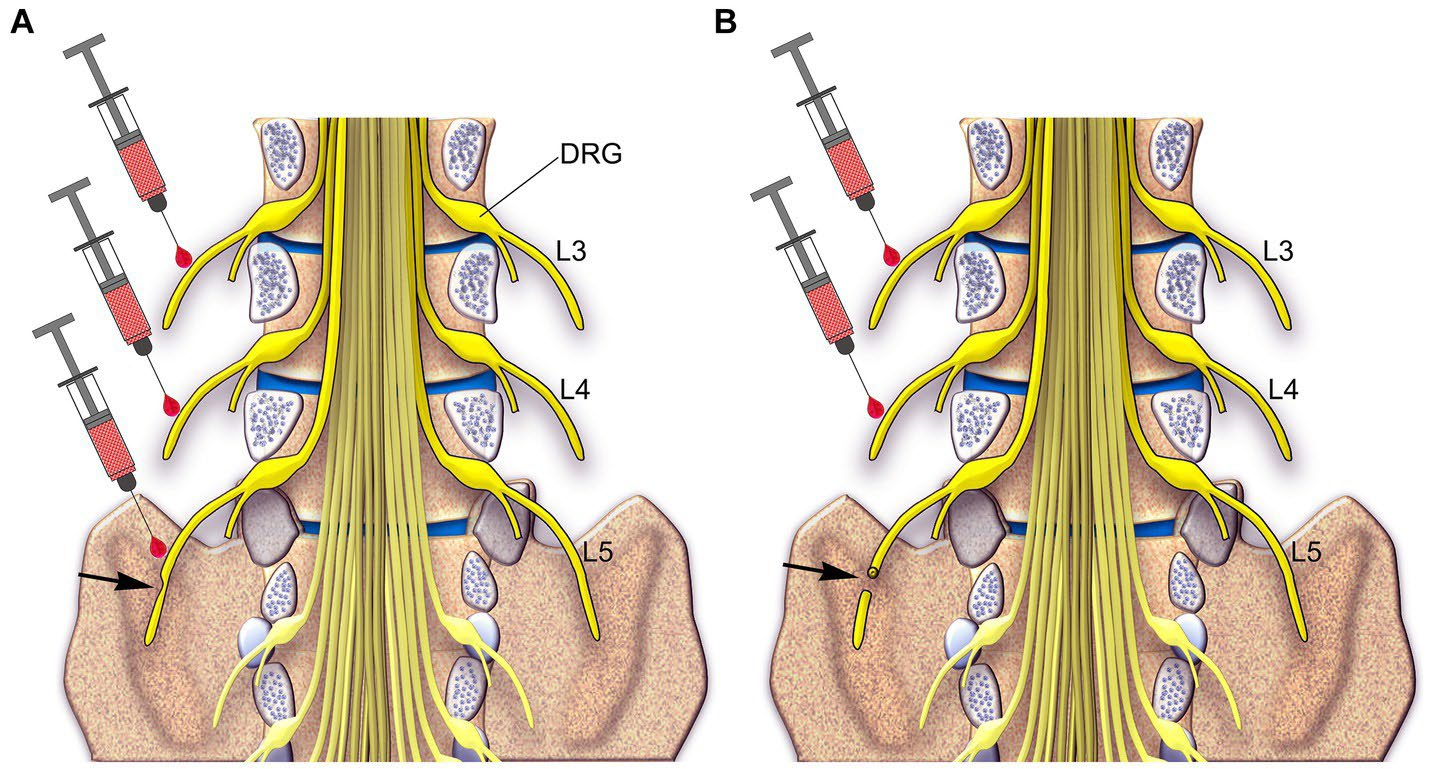
A schematic diagram showing the spinal cord inside the spinal canal and spinal nerves (yellow) in humans. Two approaches are proposed to treat and prevent neuropathic pain: Approach 1: in the partial injury of the L5 nerve (arrow in **A**), which causes neuropathic pain, injection of RTX would be carried out around the L3 and L4 uninjured nerves and the partially injured L5 nerve. Approach 2: in the complete injury of the L5 nerve (arrow in **B**), injection of RTX would be carried out around the L3 and L4 uninjured nerves.

Based on the findings of how to treat neuropathic pain in animals using local applications of capsaicin and RTX (Jancsó et al., 2007; Neubert et al., 2008; Sapio et al., 2018; Javed et al., 2020, 2022; Iadarola et al., 2021), it is now imperative to test whether the procedures developed in animals can be translated from the laboratory to the clinic to treat neuropathic pain in human patients, for whom currently available treatments are ineffective. Figure 12 summarizes our proposals on how to treat neuropathic pain following partial or complete peripheral nerve injury in patients due to trauma, post-surgery, vertebral disc herniation, nerve entrapment, ischemia, and postherpetic lesion.

## 5 Conclusion

Our study highlights the significance of the peripheral perineural application of RTX in preventing neuropathic pain subsequent to peripheral nerve injury. The action of RTX in neuropathic pain management involves the downregulation of Trpv1 and other nociceptive neurotransmitters, ion channels, and receptors associated with pain perception and transmission. The results obtained from this study and our recent research (Javed et al., 2020) emphasize the potential of RTX application on adjacent uninjured nerves as an important intervention strategy. This approach holds promise for achieving complete relief from existing peripheral nerve injury-induced neuropathic pain and preventing its further progression in both animal models and human patients affected by this debilitating condition.

## Data availability statement

The original contributions presented in the study are included in the article/Supplementary material, further inquiries can be directed to the corresponding author.

## Ethics statement

The animal study and all experimental procedures were approved by the Animal Ethics Committee of the CMHS, UAE University (ERA-2020-7222) and were performed in accordance with the guidelines of the European Communities Council Directive of November 24, 1986 (86/609/EEC). The study was conducted in accordance with the local legislation and institutional requirements.

## Author contributions

SS: Conceptualization, Data curation, Formal analysis, Funding acquisition, Investigation, Methodology, Project administration, Resources, Supervision, Validation,Writing – review & editing. HJ: Formal analysis, Methodology, Writing – original draft, Writing – review & editing. AJ: Formal analysis, Methodology, Writing – original draft. ST: Data curation, ormal analysis, Methodology, Writing – original draft. CA: Data curation, Methodology, Software, Writing – original draft. BS: Data curation, Formal analysis, Funding acquisition, Methodology, Software, Supervision, Writing – review & editing.

## Supplementary material

SUPPLEMENTARY FIGURE S1 Confocal images of a section of L4 DRG showing the localization of Kir4.1 in the satellite cells (green in A and C) surrounding large NF200-labeled neurons (Red in A and C) and unstained small-sized neurons. TpPro was used to counterstain the nuclei of the satellite cells (blue in B). Scale bar = 50 µm.

SUPPLEMENTARY FIGURE S2 Images of double immunofluorescent labeling showing Kir4.1 immunoreactivity mainly localized in the satellite cells surrounding the neurons (A, C, D, F, G and I) in the L4 DRG, including Trpv1+ neurons (B, C, E and F). RTX treatment showed a significant reduction in the number of Trpv1+ neurons (****p* < 0.001) (h) but produced no effect on the Kir4.1 immunoreactivity (G) in the L4 DRG compared to the right control (R4) and vehicle-treated L4 DRGs (A, B, D and E). Scale bar = 50 µm.
