# Correction: Unveiling the mechanisms of neuropathic pain suppression: perineural resiniferatoxin targets Trpv1 and beyond

**DOI:** 10.3389/fnana.2026.1824372

**Published:** 2026-03-27

**Authors:** Safa Shehab, Hayate Javed, Aishwarya Mary Johnson, Saeed Tariq, Challagandla Anil Kumar, Bright Starling Emerald

**Affiliations:** Department of Anatomy, College of Medicine and Health Sciences, United Arab Emirates University, Al-Ain, United Arab Emirates

**Keywords:** Trpv1, resiniferatoxin, nerve injury, ion channels, neuropathic pain

In the published article, incorrect images were erroneously included in Figure 5B, 5E. The corrected Figure 5 appears below.

**Figure 5:**
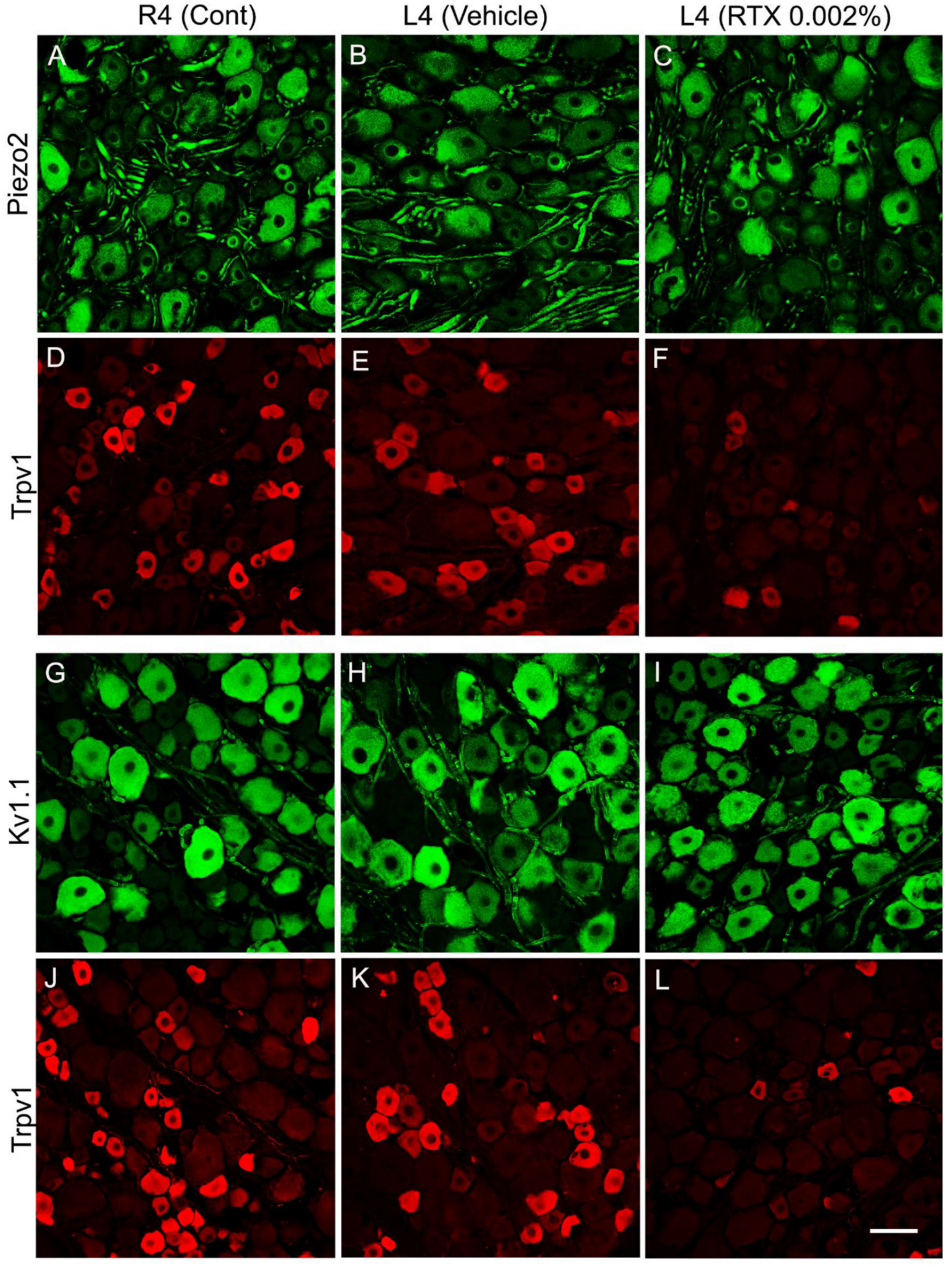
Double immunofluorescent labeling of either Piezo2 **(A–C)** and Trpv1 **(D–F)** or Kv1.1 **(G–I)** and Trpv1 **(J–L)** neurons in control right L4 DRG (R4 cont, **A, D, G, J**) and left L4 DRGs following 14 days of the perineural application of vehicle **(B, E, H, K)** or 0.002% RTX **(C, F, I, L)** on the left L4 nerve. Piezo2+ **(A–C)** and Kv1.1+ **(G–I)** immunoreactivities were mainly localized in large-sized neurons, while Trpv1+ neurons were mainly found in small-sized neurons **(D–F, J–L)** with negligible colocalization. RTX treatment showed a significant reduction in the number of Trpv1+ neurons (^***^p < 0.001) **(F, L)** but produced no effect on the Piezo2+ **(C)** or Kv1.1+ neurons **(L)** (*p* > 0.05) **(I)** in the L4 DRG compared to the right control and vehicle-treated L4 DRGs **(A, B, G, H)**. *n* = 3–4, scale bar = 50 μm.

The original version of this article has been updated.

