# Supplementary figures and images for: Unveiling the mechanisms of neuropathic pain suppression: perineural resiniferatoxin targets Trpv1 and beyond
Source: Front Neuroanat. 2023 Nov 30;17:1306180. doi: 10.3389/fnana.2023.1306180 (PMC10720729; doi:10.3389/fnana.2023.1306180)

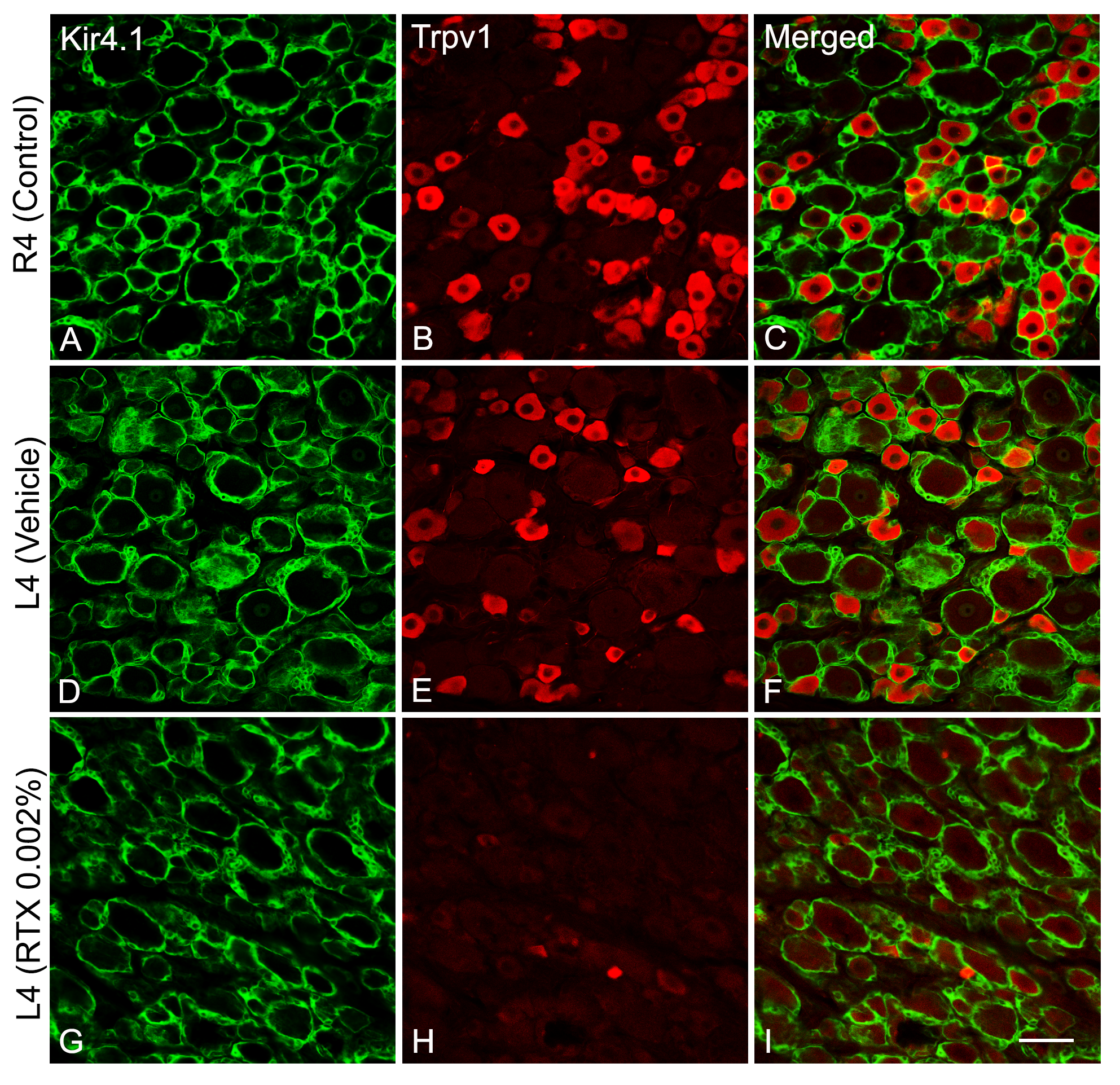

Supplement: SUPPLEMENTARY FIGURE S1 — Confocal images of a section of L4 DRG showing the localization of Kir4.1 in the satellite cells (green in A and C) surrounding large NF200-labeled neurons (Red in A and C) and unstained small-sized neurons. TpPro was used to counterstain the nuclei of the satellite cells (blue in B). Scale bar = 50 µm. [file Image_1.TIF]

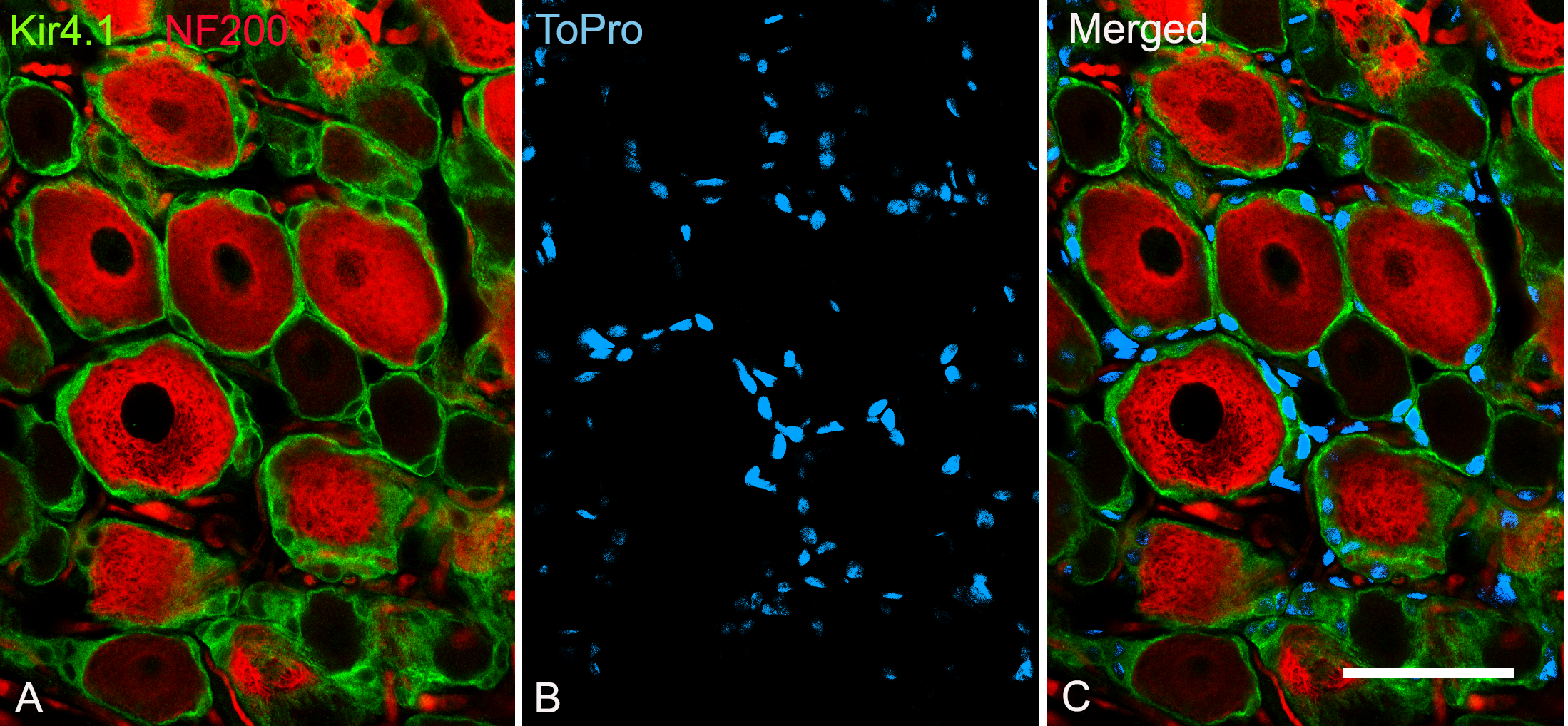

Supplement: SUPPLEMENTARY FIGURE S2 — Images of double immunofluorescent labeling showing Kir4.1 immunoreactivity mainly localized in the satellite cells surrounding the neurons (A, C, D, F, G and I) in the L4 DRG, including Trpv1+ neurons (B, C, E and F). RTX treatment showed a significant reduction in the number of Trpv1+ neurons (***p < 0.001) (h) but produced no effect on the Kir4.1 immunoreactivity (G) in the L4 DRG compared to the right control (R4) and vehicle-treated L4 DRGs (A, B, D and E). Scale bar = 50 µm. [file Image_2.TIF]
